# Supplementary material for: Single crystalline quaternary sulfide nanobelts for efficient solar-to-hydrogen conversion
Source: Nat Commun. 2020 Oct 15;11:5194. doi: 10.1038/s41467-020-18679-z (PMC7567062; doi:10.1038/s41467-020-18679-z)
Supplement: Supplementary file 1 — Supplementary Information [file 41467_2020_18679_MOESM1_ESM.docx]

Supplementary Information

**Single crystalline quaternary sulfide nanobelts for efficient solar-to-hydrogen conversion**

Wu et al.


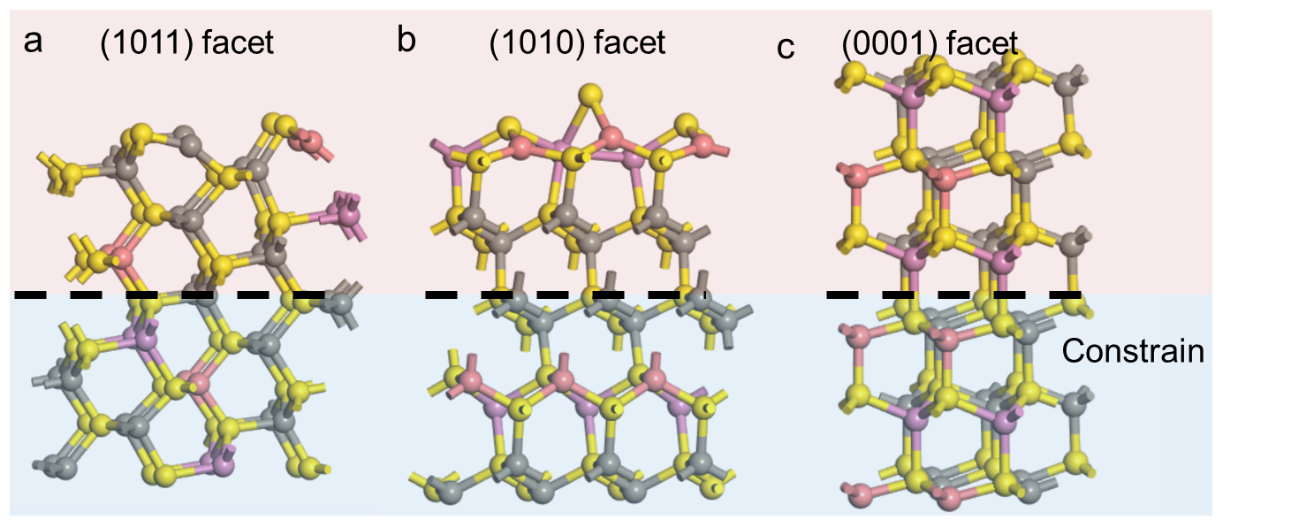


**Supplementary Figure 1 |** **Crystal facets of CZIS which is used for simulation**. **a**, (1011) facet. **b**, (1010) facet. **c**, (0001) facet.


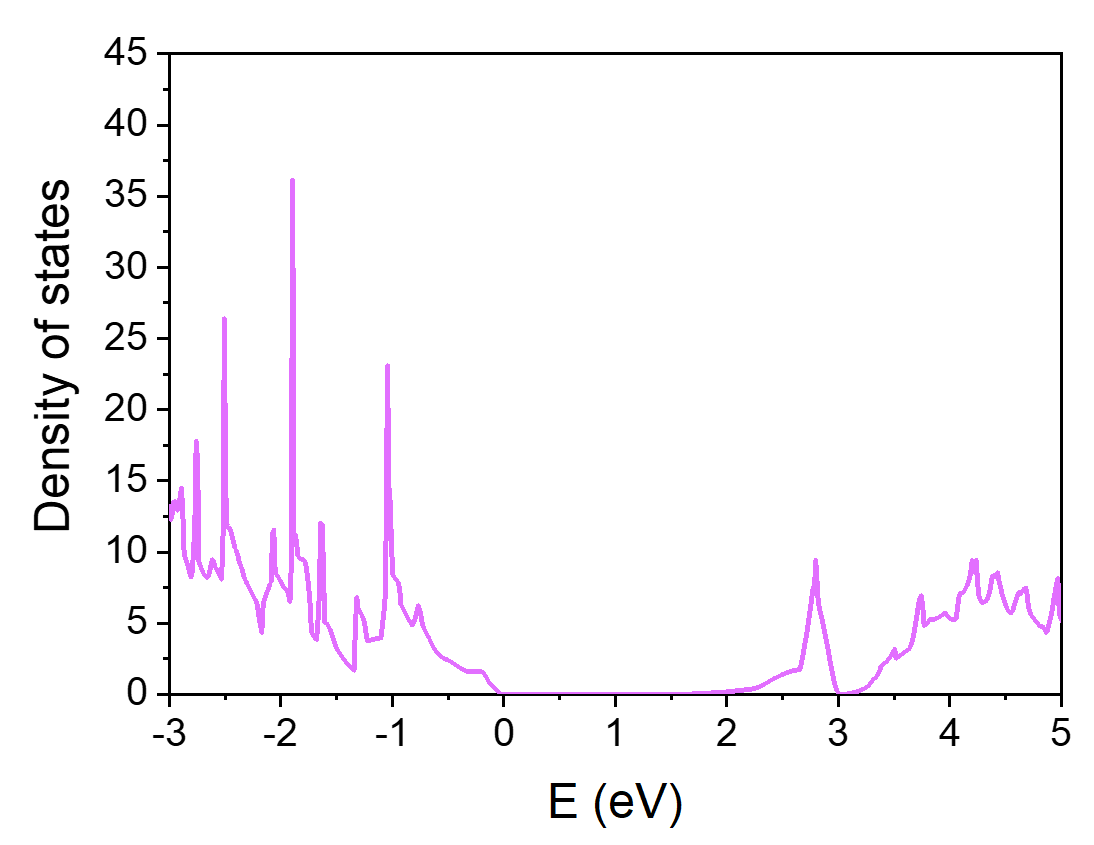


**Supplementary Figure 2 | Total density of states of CZIS calculated using HSE.**


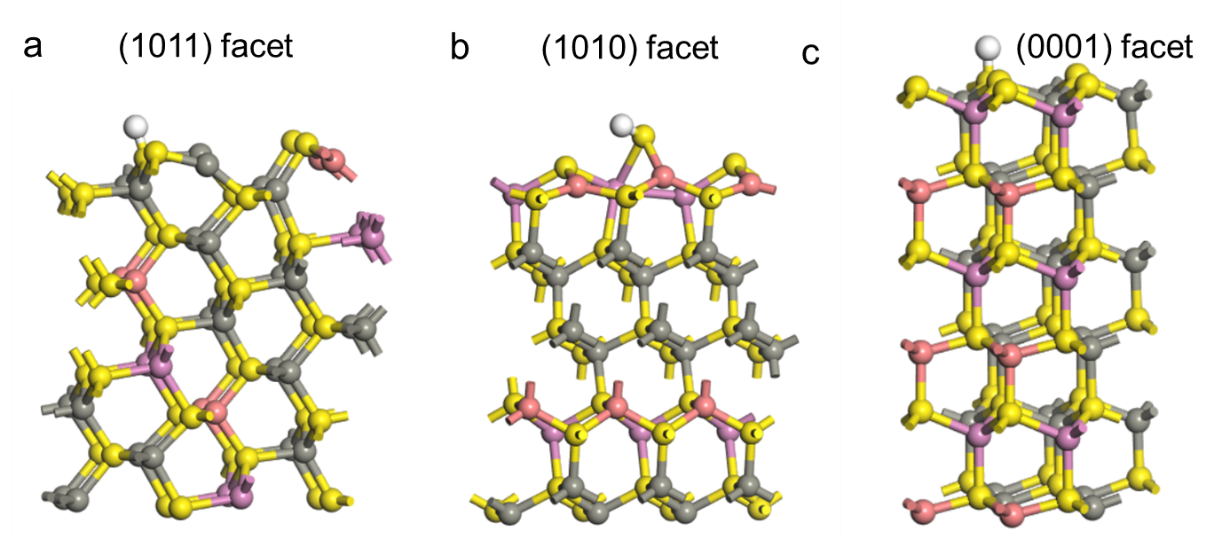


**Supplementary Figure 3 | The adsorption sites of H atom. a** (1011) facet. **b**, (1010) facet. **c**, (0001) facet.


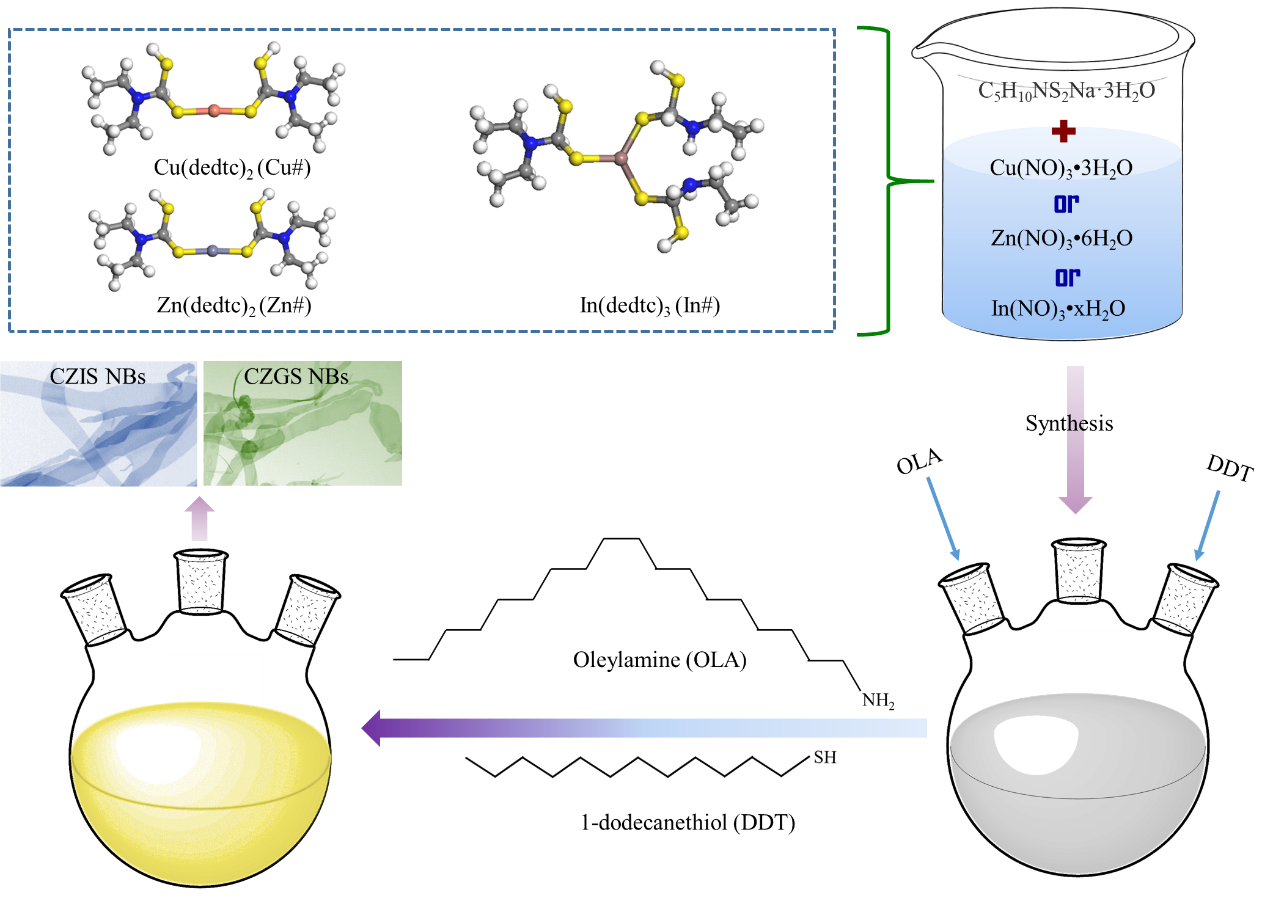


**Supplementary Figure 4 |** **Scheme of colloidal synthesis of nonlayered quaternary NBs using OLA and DDT**.


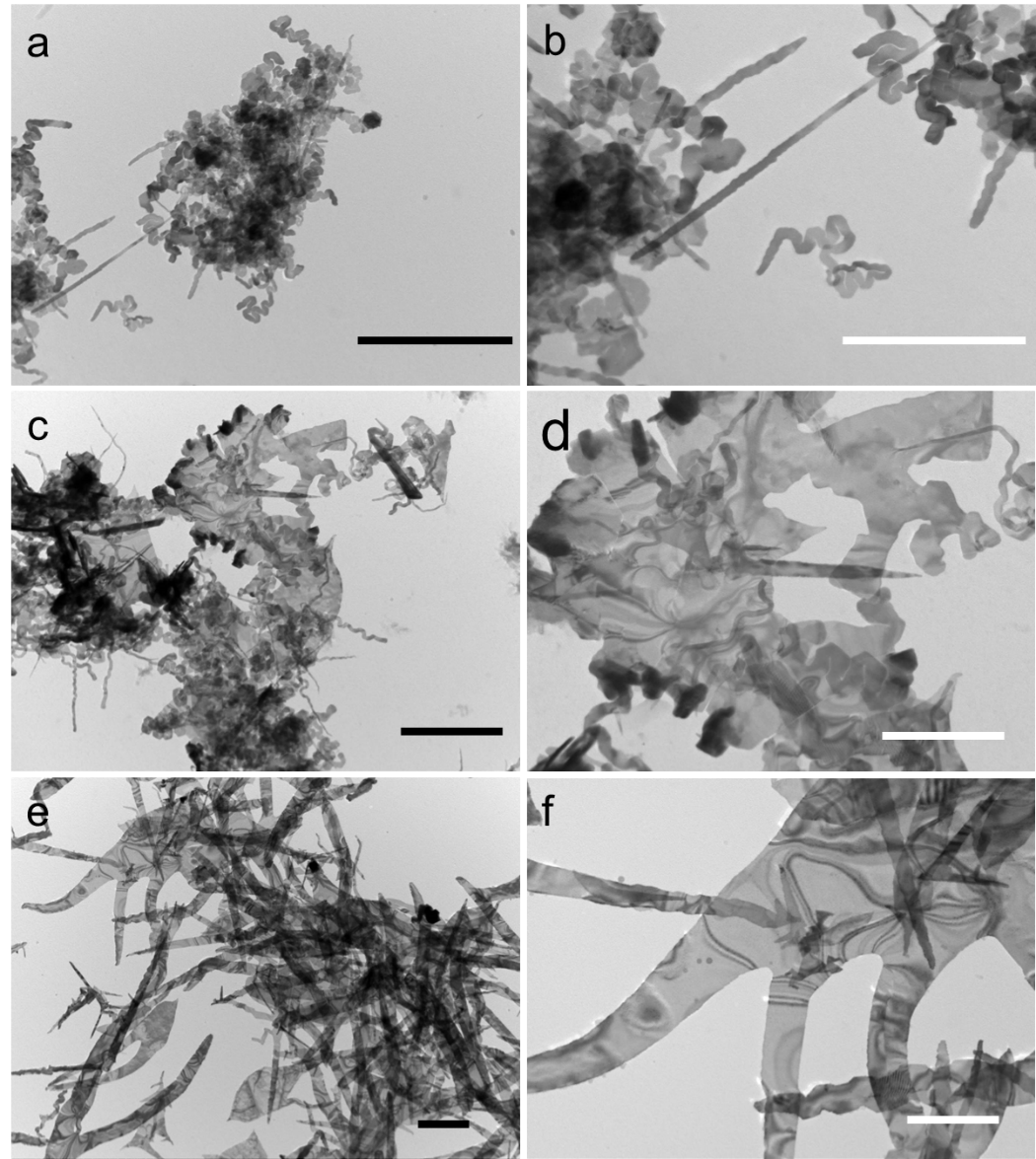


**Supplementary Figure 5 |** **TEM images of CZIS NBs synthesized at different reaction temperature**. **a-b,** 190 ^o^C. **c-d,** 220 ^o^C. **e-f,** 270 ^o^C. Scale bars are 1 μm for **a**, **c** and **e,** 500 nm for **b**, **d** and **f**, respectively.


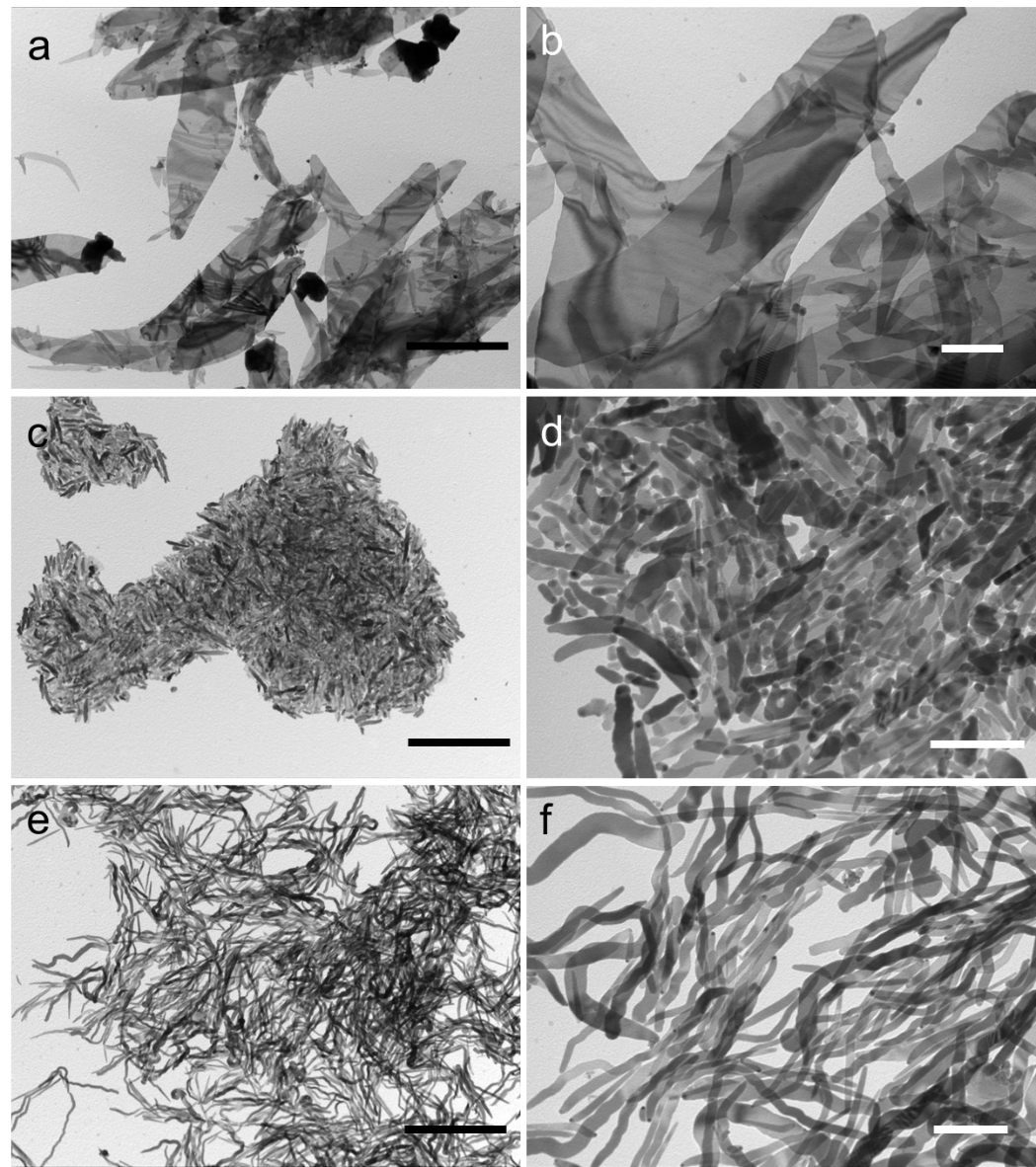


**Supplementary Figure 6 |** **TEM images of CZIS NBs synthesized with different volume of OLA, DDT and ODE.** **a-b,** 4 mL of OLA, 8 mL of DDT, 4mL of ODE. **c-d,** 8 mL of OLA, 4 mL of DDT, 4mL of ODE. **e-f,** 8 mL of OLA, 8 mL of DDT, 0mL of ODE. Scale bars are 1 μm for **a**, **c** and **e,** 200 nm for **b**, **d** and **f**, respectively.


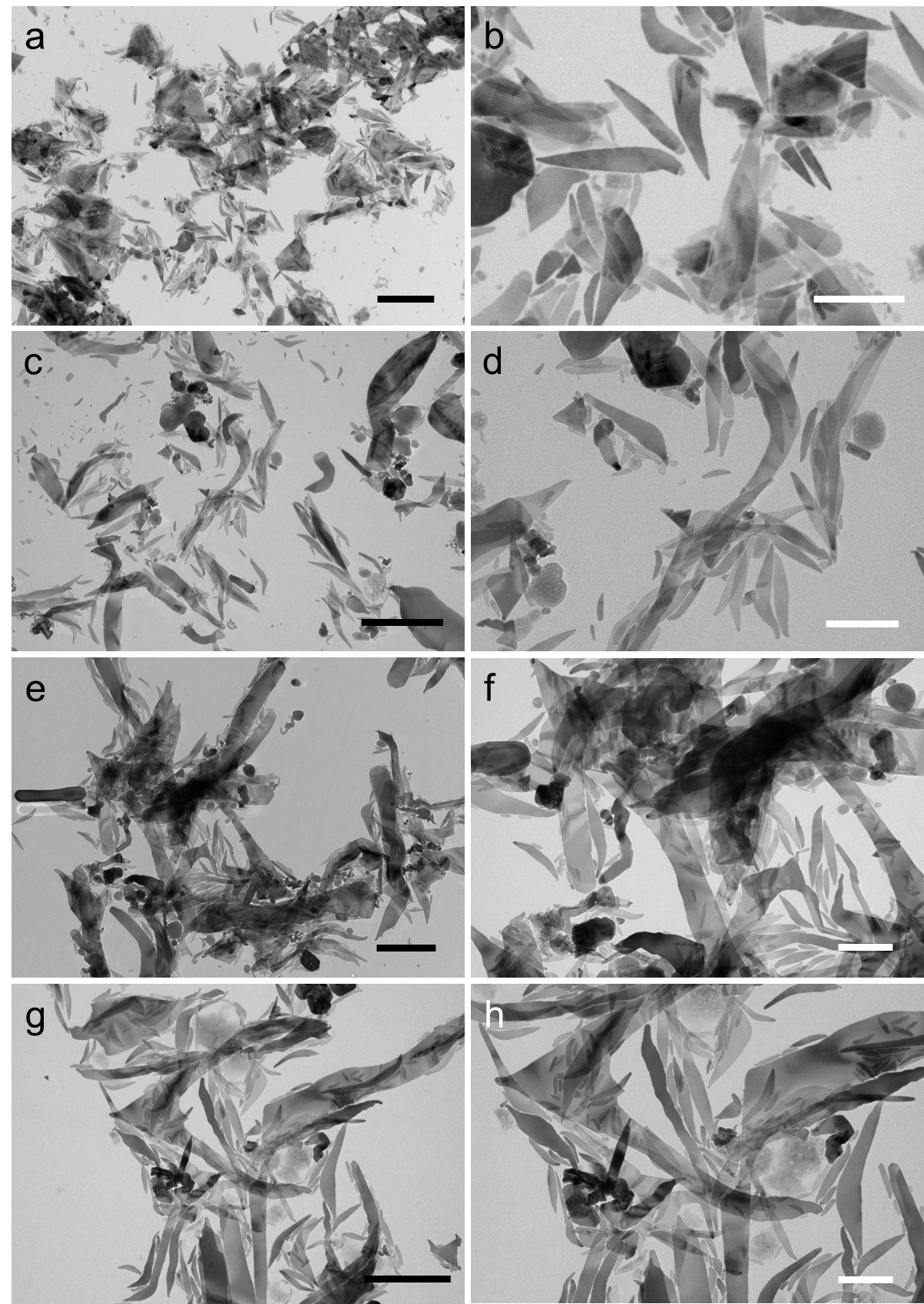


**Supplementary Figure 7 |** **TEM images of CZIS NBs synthesized with different reaction time at 250 ^o^C.** **a-b,** 0 min. **c-d,** 15 min. **e-f,** 30 min. **g-h,** 45 min. Scale bars are 500 nm for **a**, **c**, **e** and **g,** 200 nm for **b**, **d**, **f and h**, respectively.


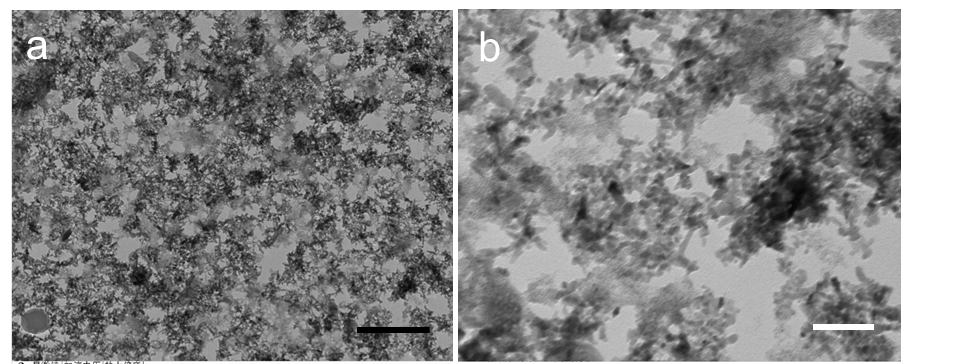


**Supplementary Figure 8 |** **TEM image (a) and enlarged TEM image (b) of the CZIS nanocrystals synthesized using OA to instead of DDT**. Scale bar are 500 nm for **a**, 100 nm for **b**, respectively.


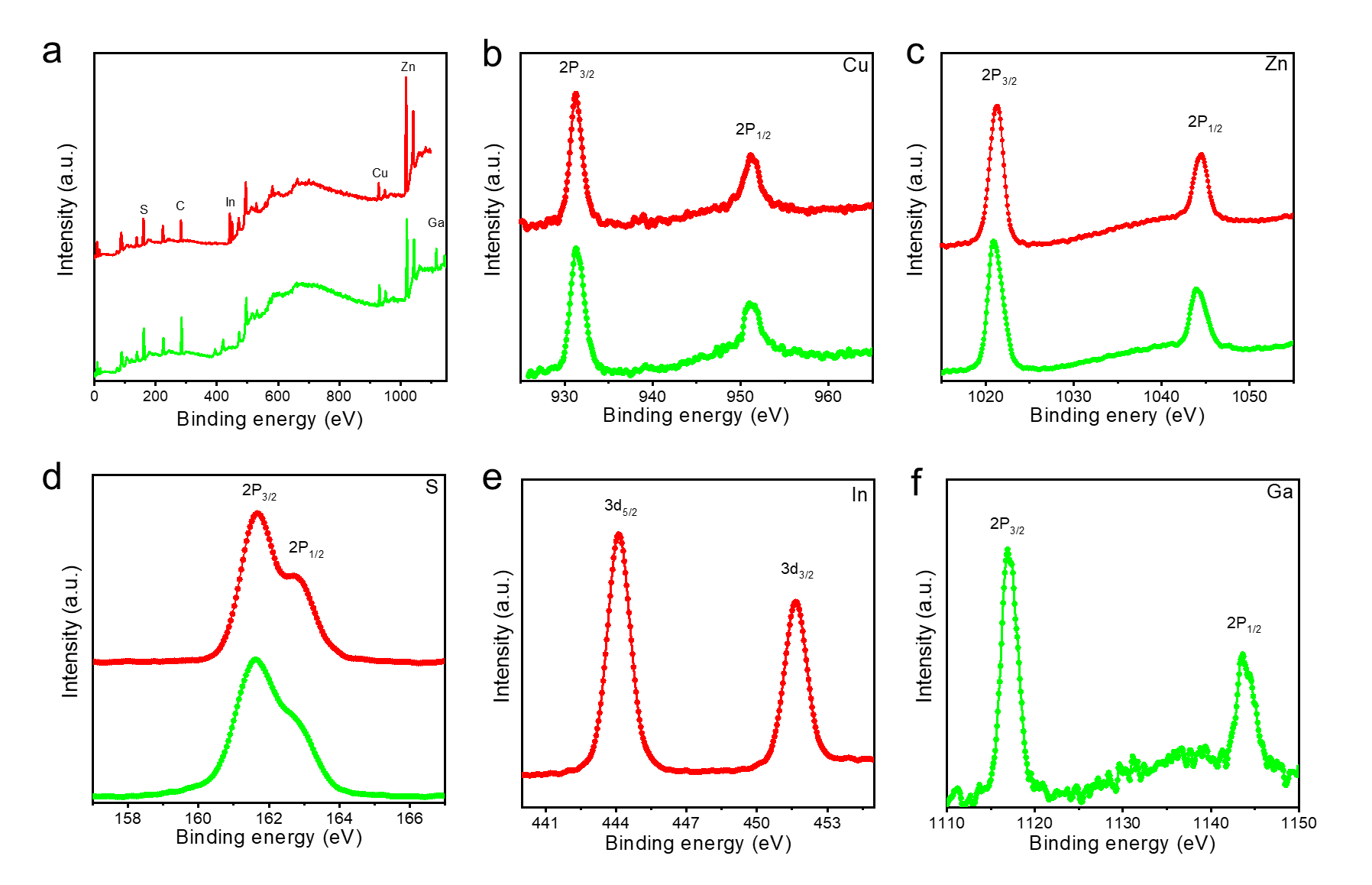


**Supplementary Figure 9 |** **XPS spectra of the synthesized wurtzite NBs.** **a,** The survey spectrum shows the presence of Cu, In, Zn, S and Cu, In, Zn, S in the CZIS and CZGS NBs, respectively. **b,** The Cu2p spectra of CZIS and CZGS NBs show two peaks, which all located at 932.2 and 952 eV with a binding energy splitting of 19.8 eV, indicating the presence of the Cu(I) state. **c,** The two peaks at 1021.4 and 1044.3 eV with a binding energy splitting of 22.9 eV confirms the existence of Zn(II) state. **d,** The typical S2p peaks at 161.9 and 163 eV can be index to the peaks of the S(II) state. **e,** The In3d spectrum of CZIS NBs confirms the presence of In(III) state. **f,** The Ga2p spectrum confirms the presence Ga(III) state. Thus, the results of XPS analysis indicate that the phase and composition cannot inﬂuence the element oxidation state of the obtained nanocrystals.


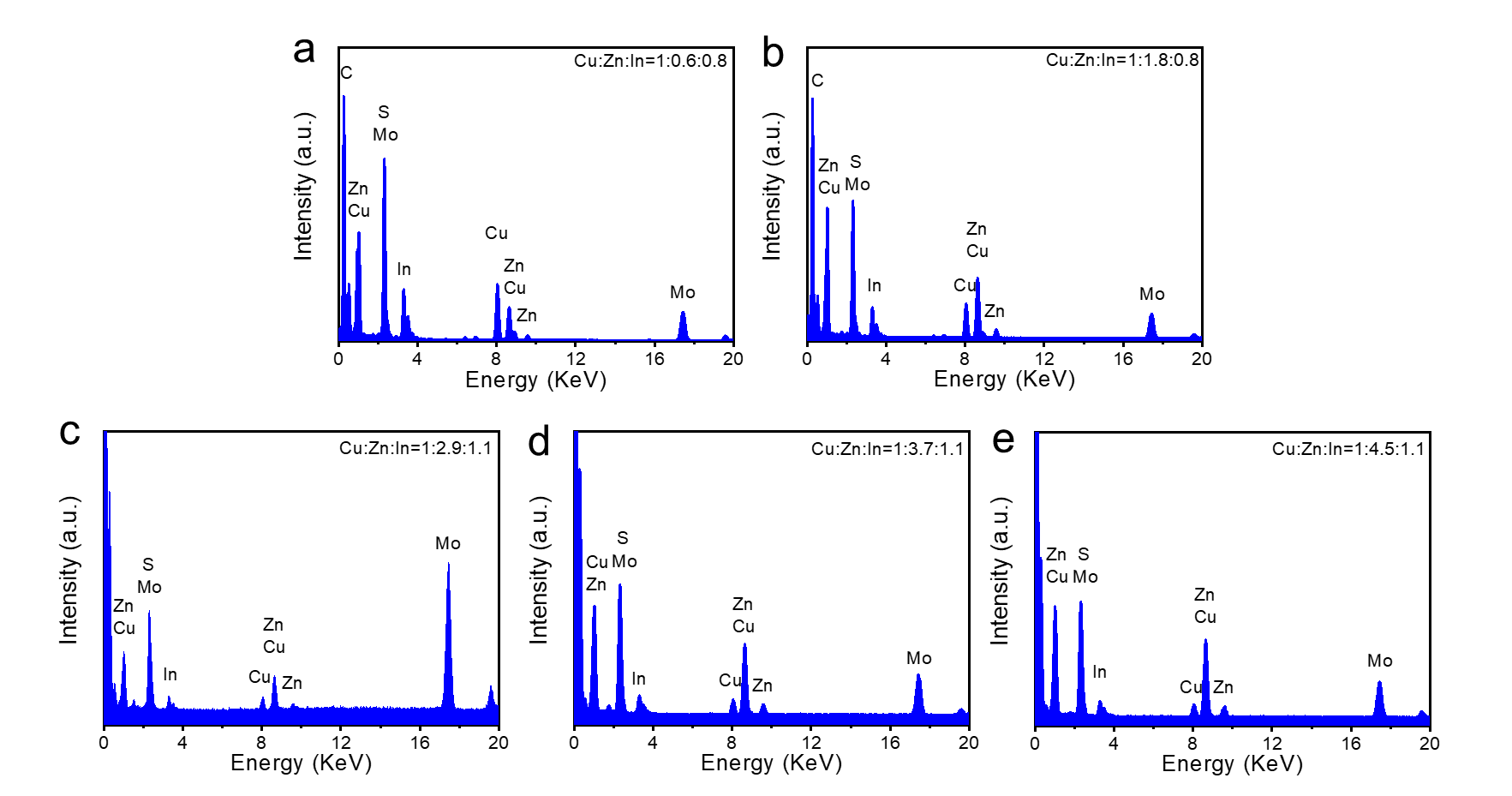


**Supplementary Figure 10 |** **The EDS spectra of the wurtzite CZIS NBs synthesized with different amounts of Zn#**. **a,** 0.2 mmol. **b,** 0.4 mmol. **c,** 0.6 mmol. **d,** 0.6 mmol. **e,** 1.0 mmol.


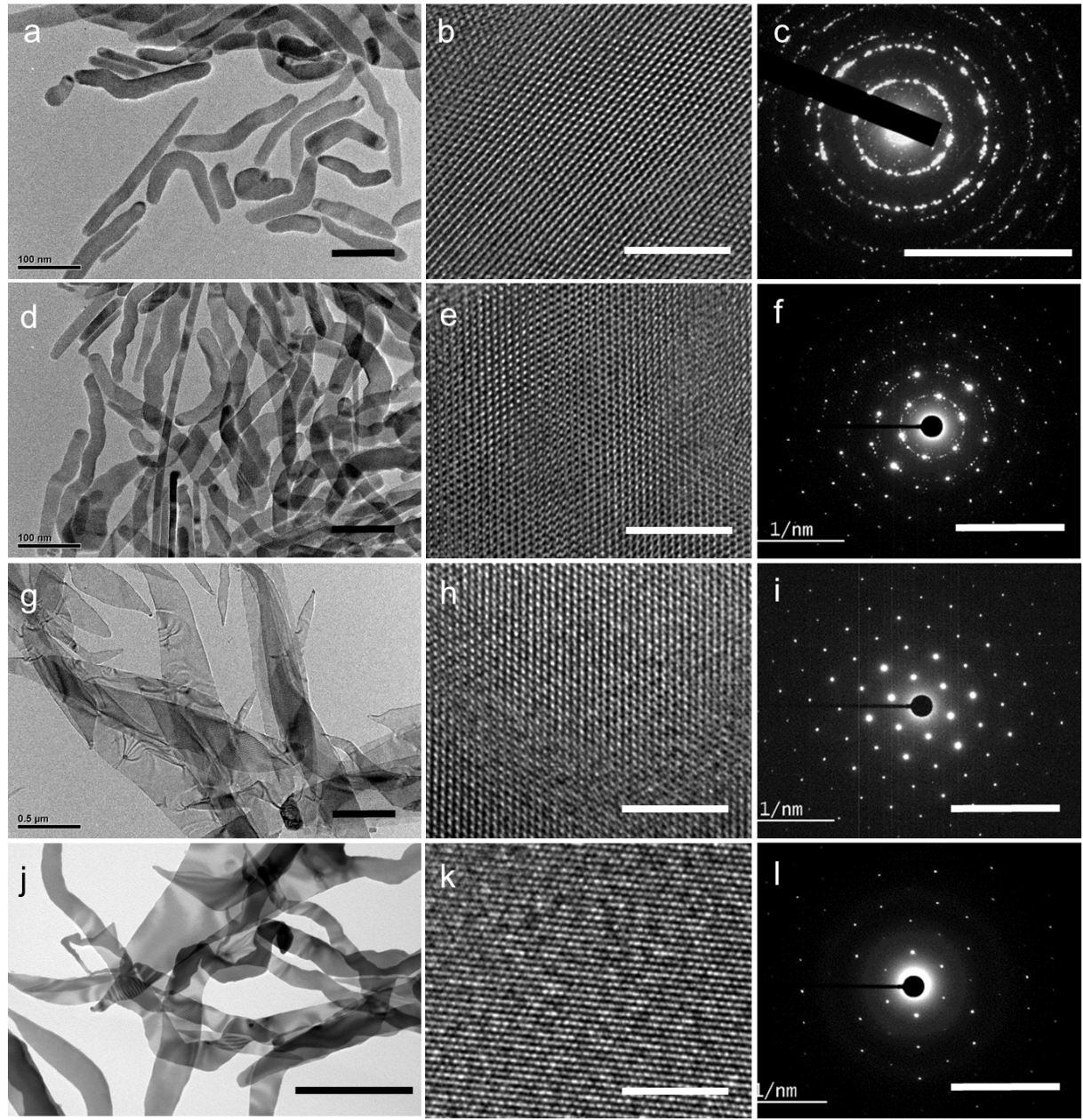


**Supplementary Figure 11 |** **TEM and HRTEM images and SAED patterns of the CZIS NBs synthesized with different amounts of Zn#.** **a-c,** 0.2 mmol. **d-f,** 0.4 mmol. **g-i,** 0.6 mmol. **j-l,** 1.0 mmol. Scale bar are 500 nm for **a**, **d**, **g** and **j**, 5 nm for **b**, **e**, **h** and **k**, 10 1/nm for **c**, **f**, **i** and **l**, respectively.


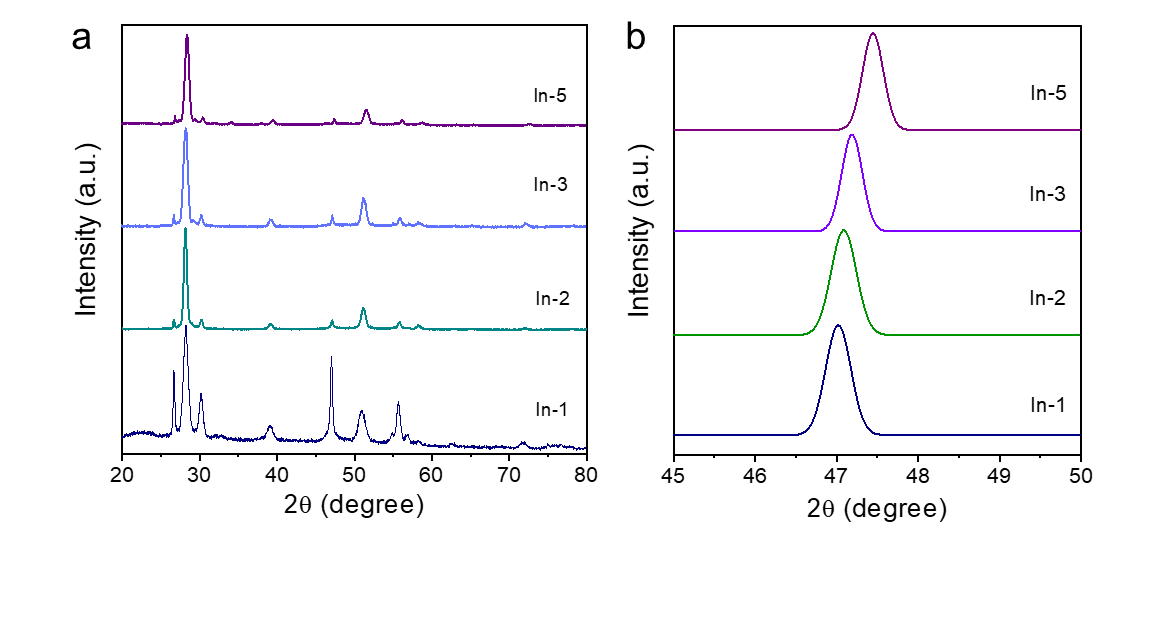


**Supplementary Figure 12 |** **Structure characterization of the wurtzite CZIS NBs synthesized with different amounts of Zn#**. **a**, PXRD pattens. **b**, the enlarged PXRD patterns.


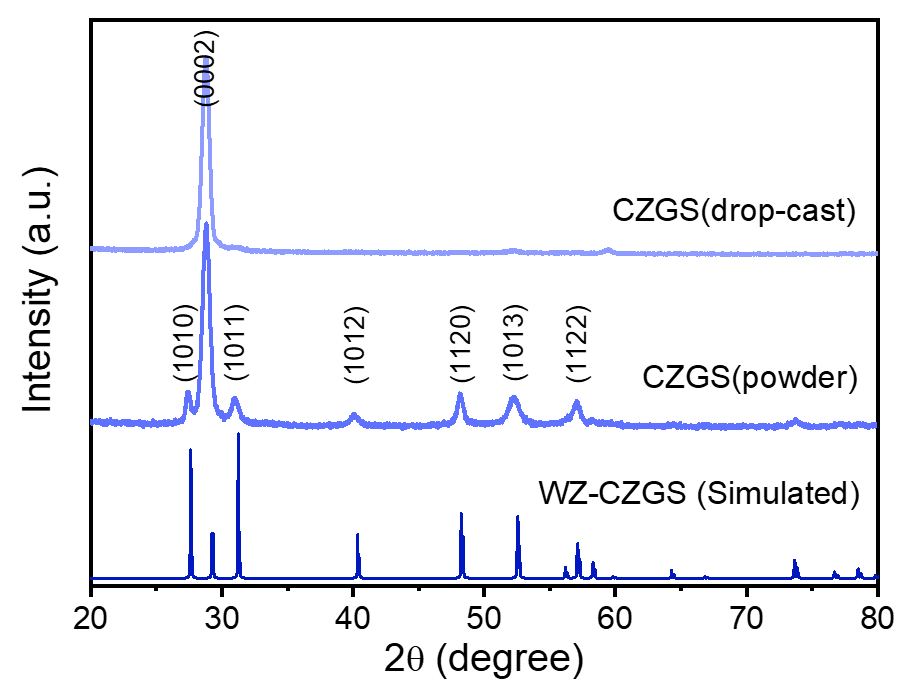


**Supplementary Figure 13 |** **XRD patterns of the obtained CZGS NBs.** For reference, the simulated wurtzite XRD pattern of CZGS is shown below. The results show a typical hexagonal wurtzite structure diffraction with exposed (0001) facet.


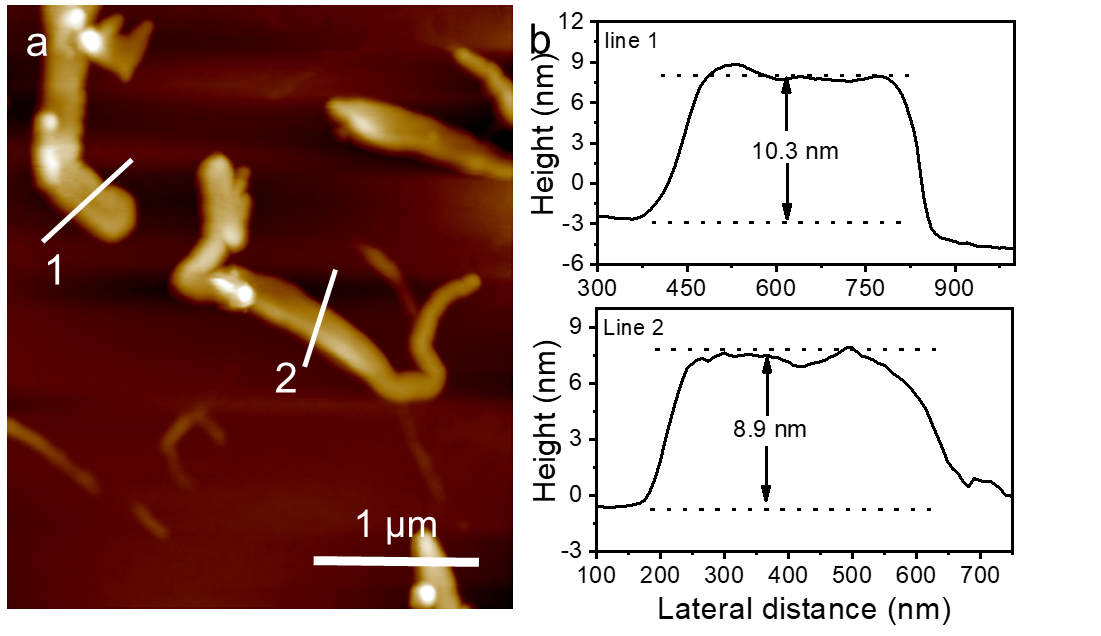


**Supplementary Figure 14 |** **Quantitating the thickness of the single crystalline wurtzite CZGS NBs**. **a-b,** AFM image and corresponding height images . Scale bar is 1 μm for **a**.


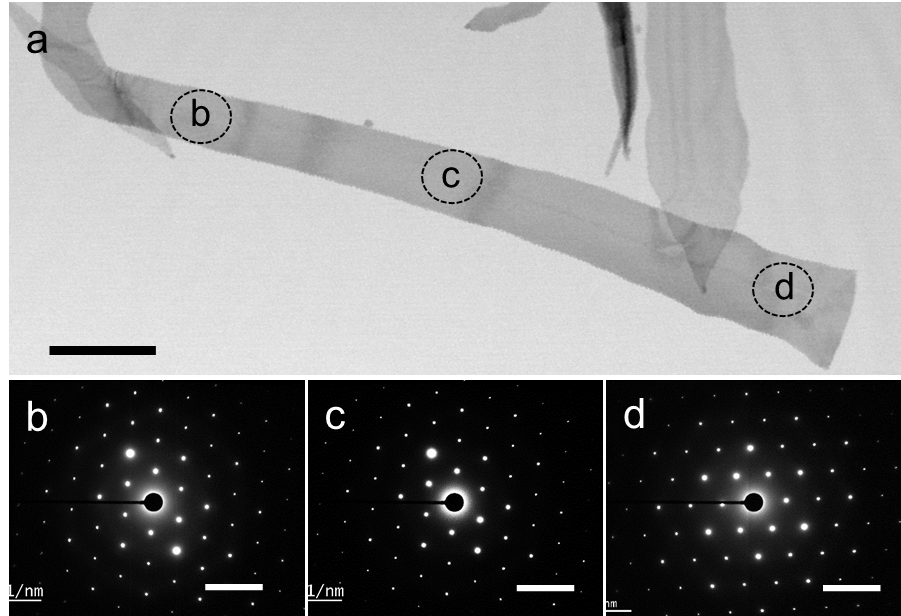


**Supplementary Figure 15 |** **Characterization of a typical CZGS nanobelt**. **a,** TEM image. **b-d,** the SAED patterns of the areas in **a**. Scale bars are 200 nm for **a**, 10 1/nm for **b**, **c** and **d**, respectively.


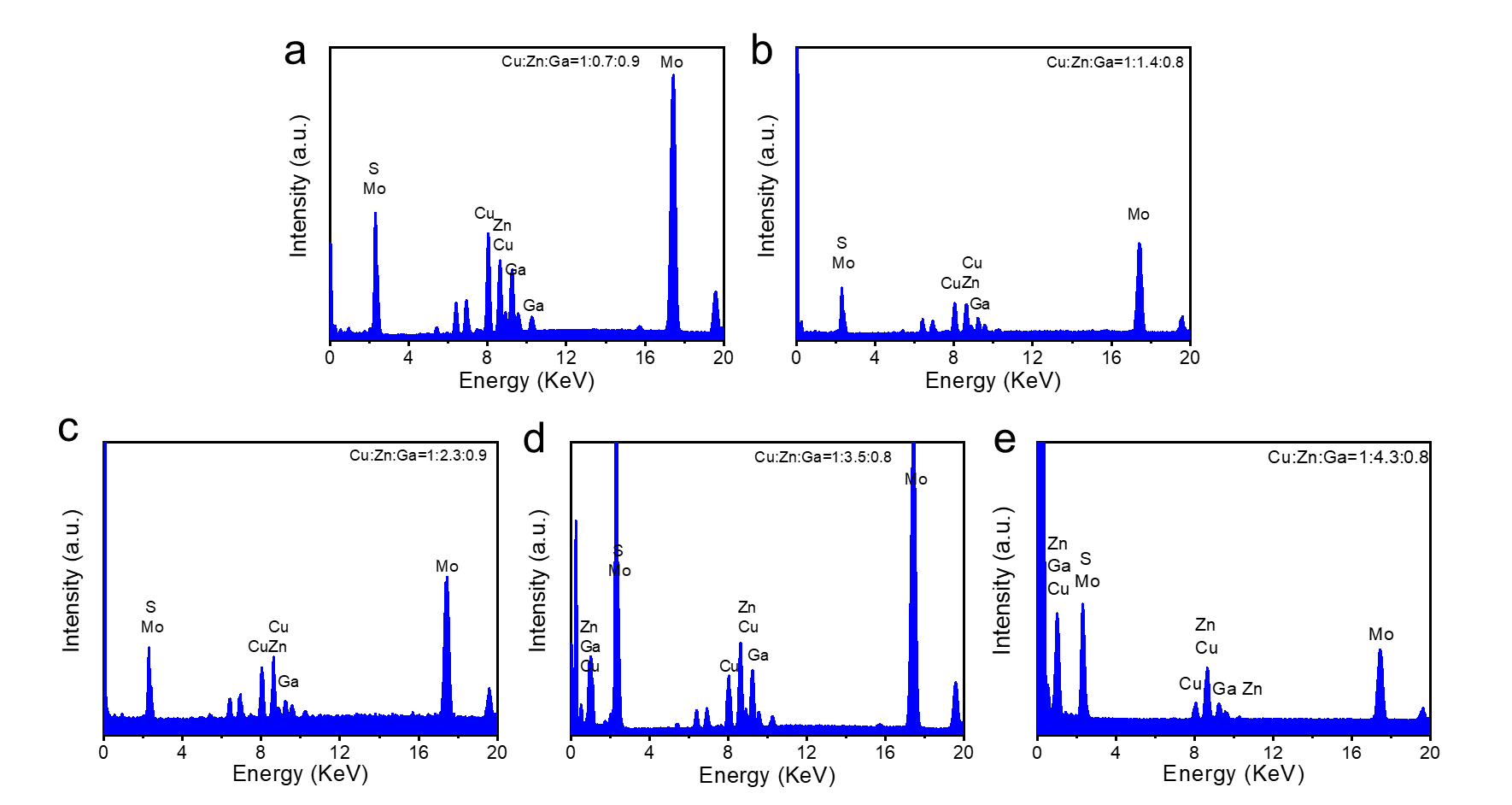


**Supplementary Figure 16 |** **The EDS spectra of the wurtzite CZGS NBs synthesized with different amounts of Zn#**. **a,** 0.2 mmol. **b,** 0.4 mmol. **c,** 0.6 mmol. **d,** 0.6 mmol. **e,** 1.0 mmol.


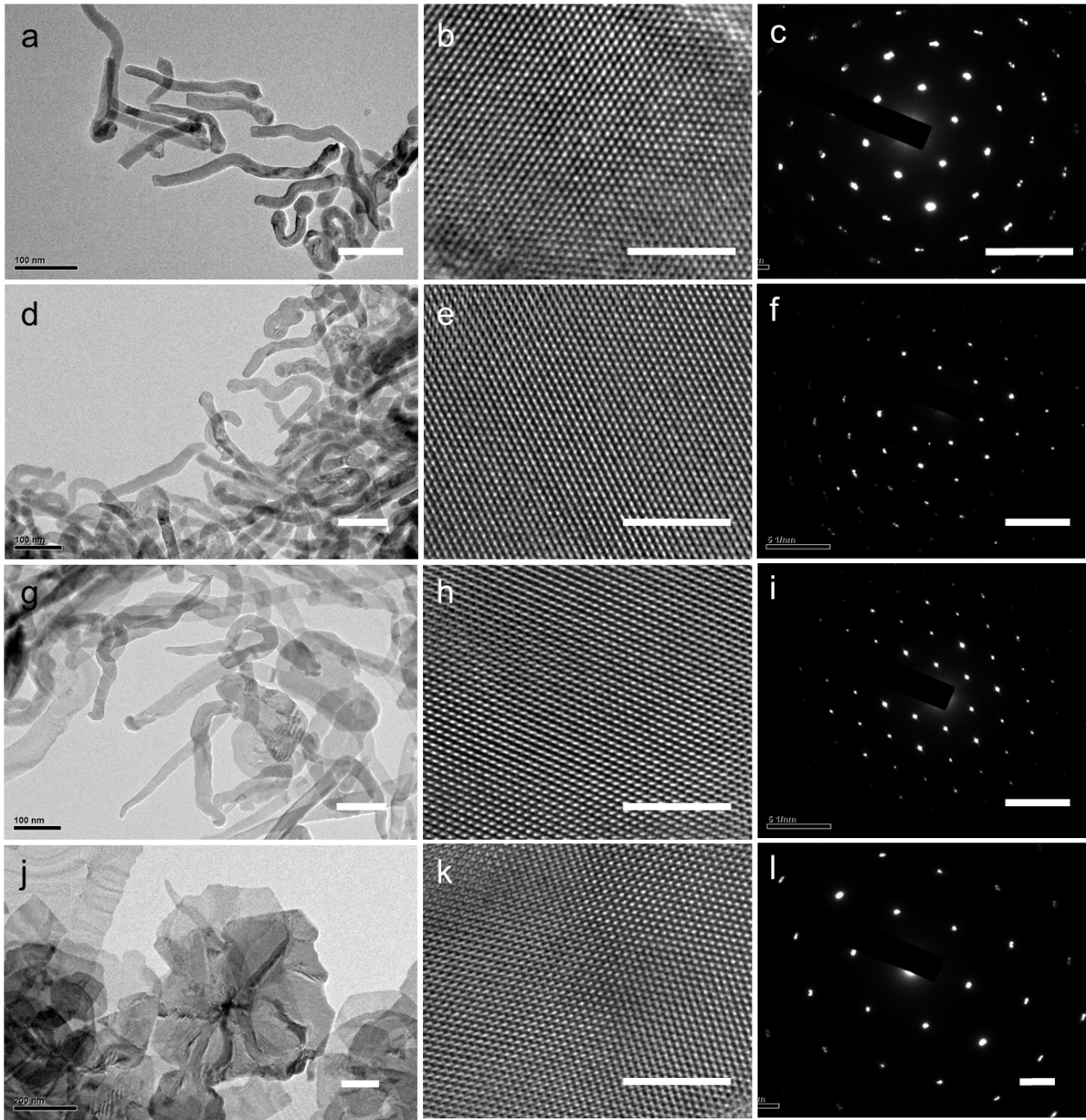


**Supplementary Figure 17 |** **TEM and HRTEM images and SAED patterns of the CZGS NBs synthesized with different amounts of Zn#. a-c,** 0.2 mmol. **d-f,** 0.4 mmol. **g-i,** 0.6 mmol. **j-l,** 1.0 mmol. Scale bar are 100 nm for **a**, **d**, **g** and **j**, 5 nm for **b**, **e**, **h** and **k**, 10 1/nm for **c**, **f**, **i** and **l**, respectively.


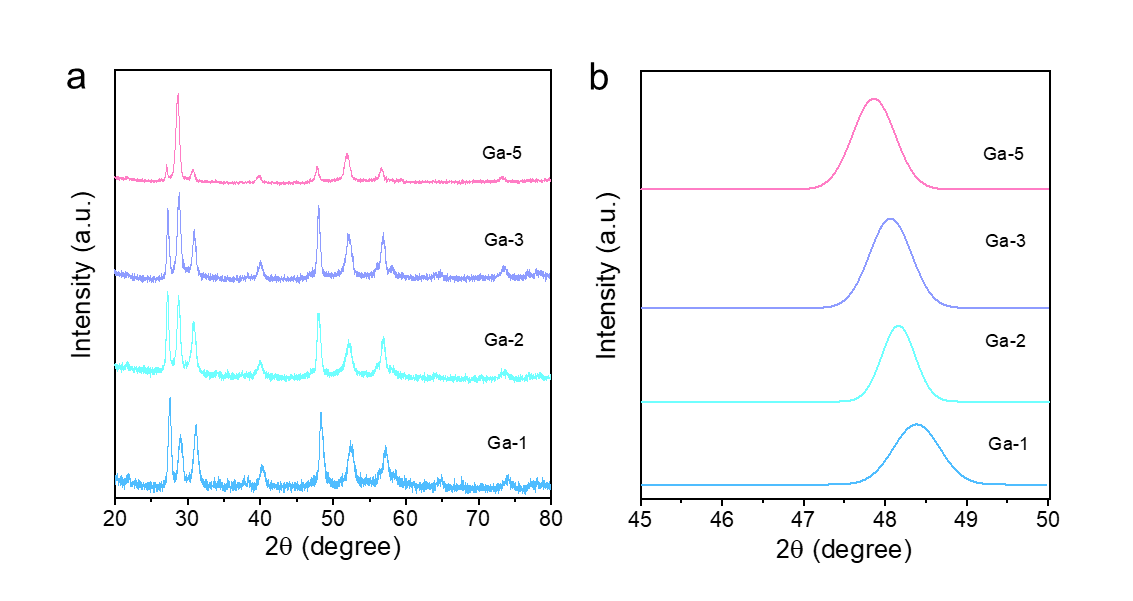


**Supplementary Figure 18 |** **Structure characterization of the wurtzite CZGS NBs synthesized with different amounts of Zn#**. **a**, PXRD pattens. **b**, the enlarged PXRD patterns.


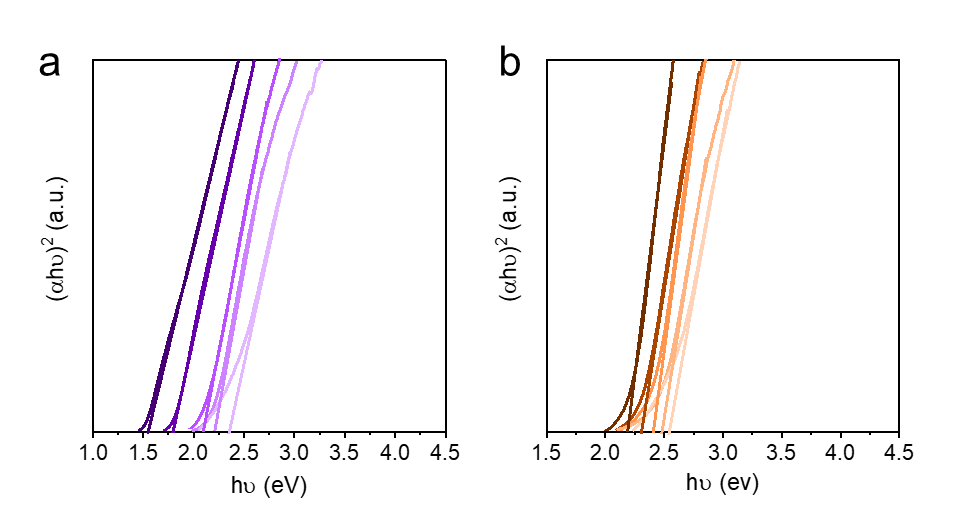


**Supplementary Figure 19 |** **The graph of linear extrapolation of (αhυ)^2^ versus photon energy (hυ)**. **a**, the CZIS NBs. **b**, CZGS NBs.


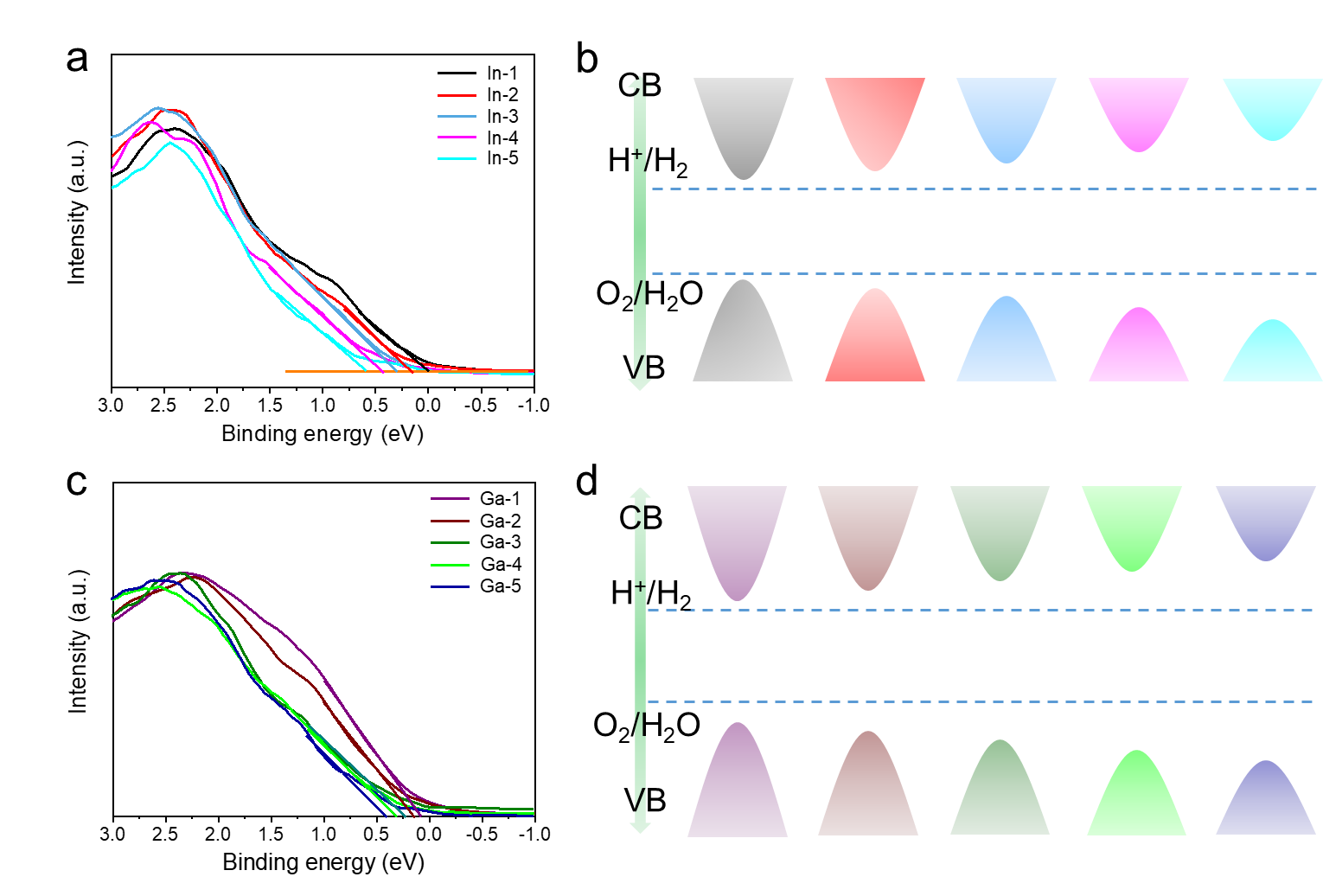


**Supplementary Figure 20 | Band structure of the synthesize NBs. a** and **c**, Valence band (VB) XPS spectra of the synthesized wurtzite CZIS and CZGS NBs. **b,** Band structure of CZIS NBs between ZnS and CuInS_2_. **d,** Band structure of CZGS NBs between ZnS and CuGaS_2_.


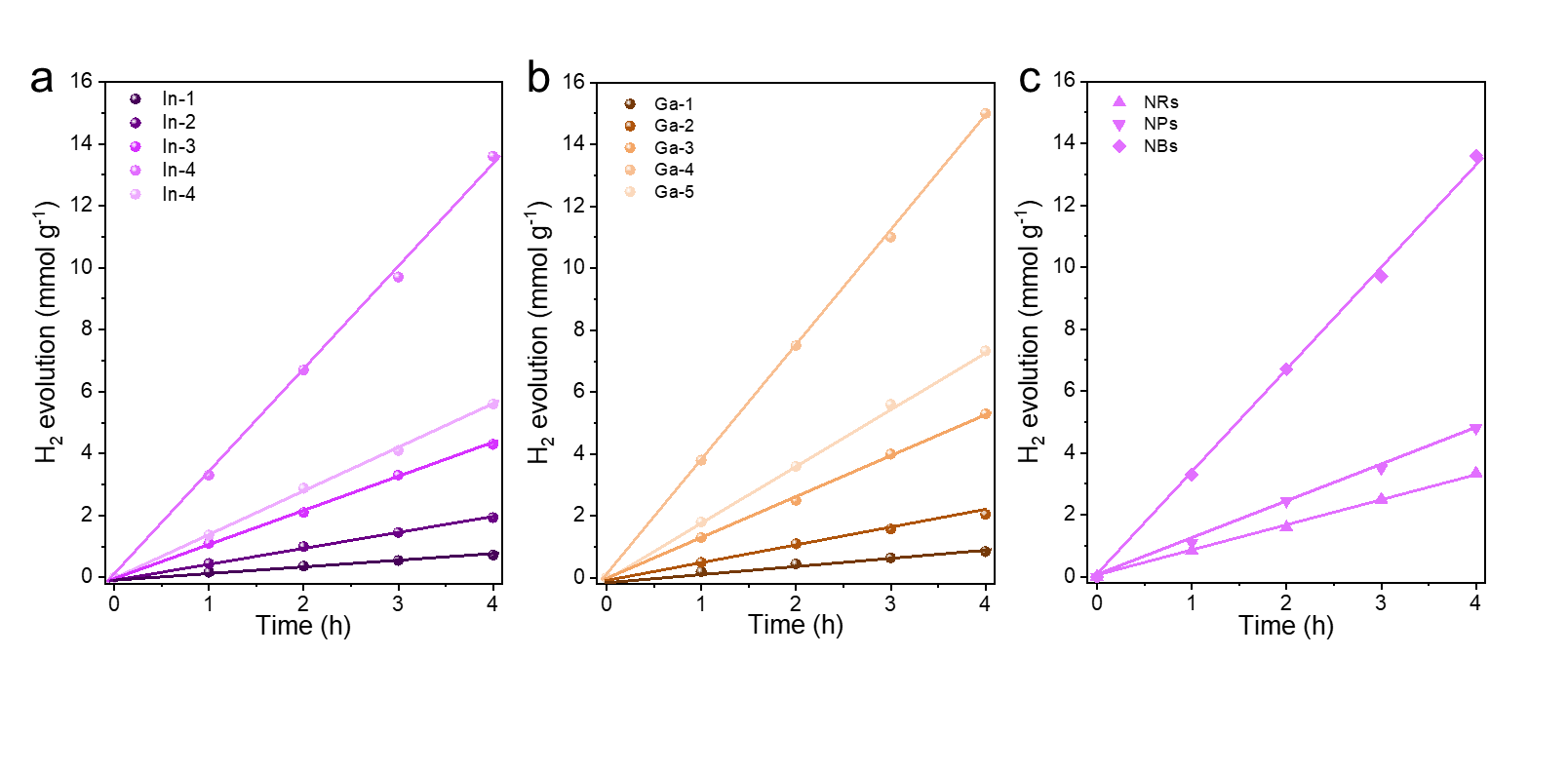


**Supplementary Figure 21 | Comparison photocatalytic H_2_ activities.** **a,** CZIS NBs with different Zn contents. **b,** CZGS NBs with different contents. **c,** CZIS nanorods, nanoparticles and nanobelts.


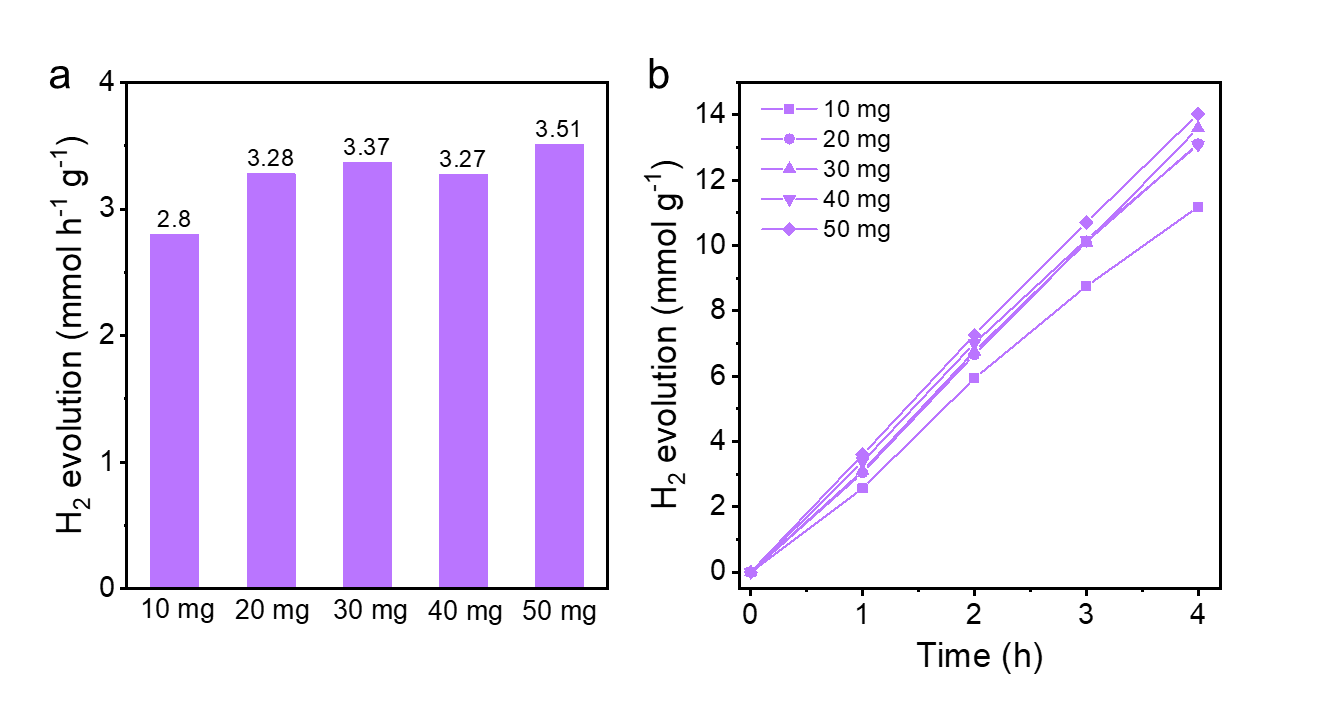


**Supplementary Figure 22 | Photocatalytic performances of the CZIS nanobelts with different amounts** **under visible-light irradiation (λ ˃ 420 nm). a**, Comparison of hydrogen evolution rates. **b**, Comparison of hydrogen evolution activities.


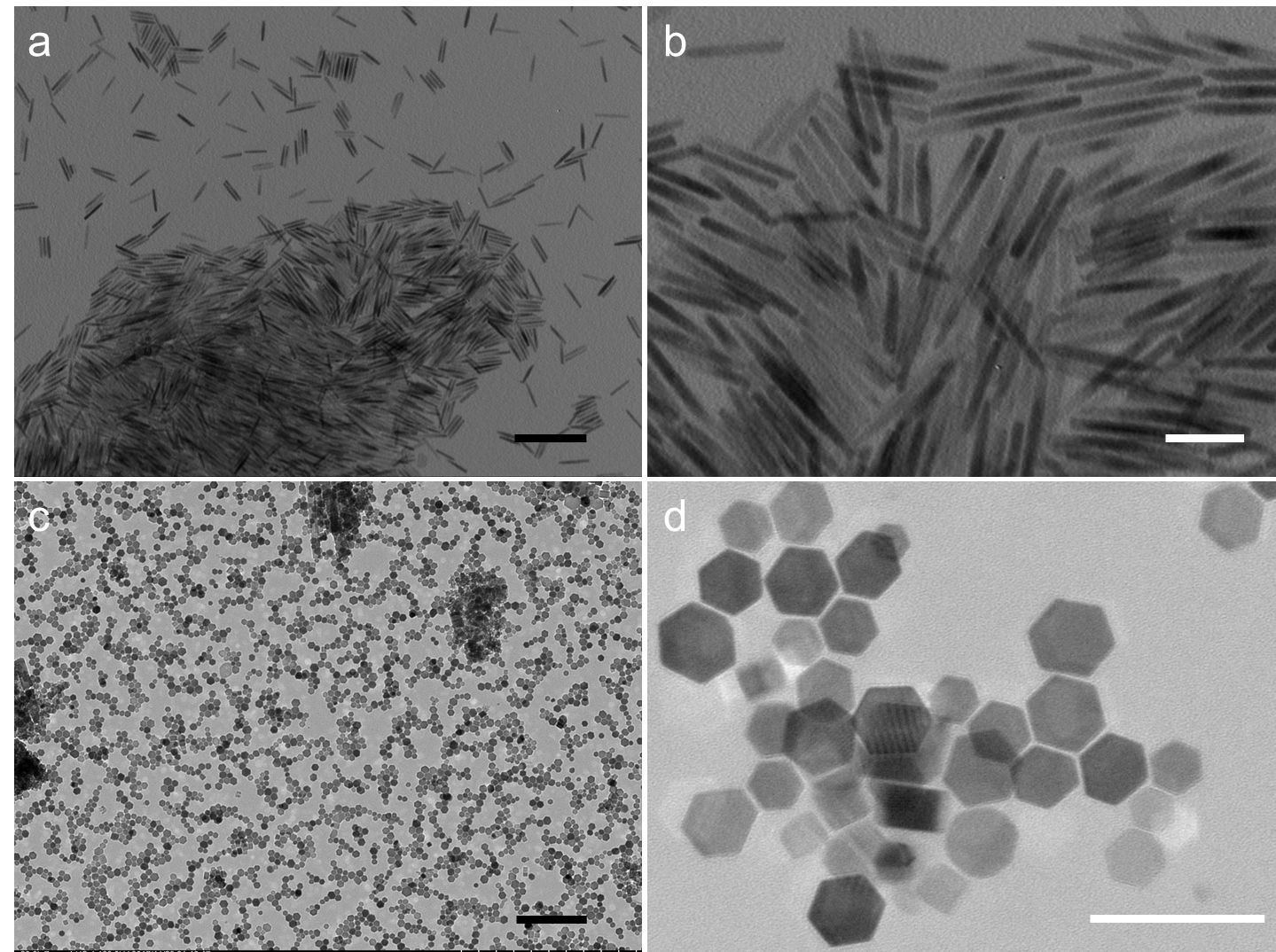


**Supplementary Figure 23 |** **Characterization of the synthesized CZIS nanorods and nanoparticles**. **a-b,** TEM and enlarged TEM images of CZIS nanorods. **c-d,** TEM and enlarged TEM images of CZIS nanoparticles. Scale bars are 200 nm for **a** and **c**, 50 nm for **b** and **d**, respectively.


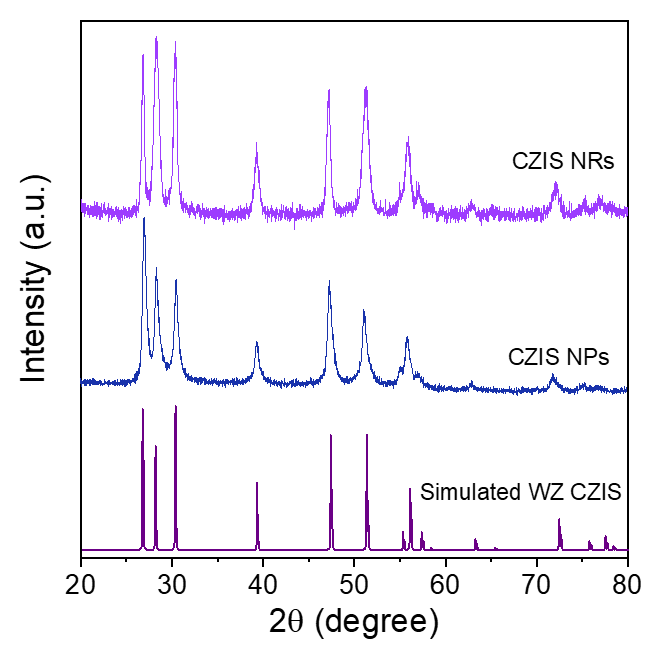


**Supplementary Figure 24 |** **The PXRD patterns of CZIS nanorods (NRs) and nanoparticles (NPs)**.


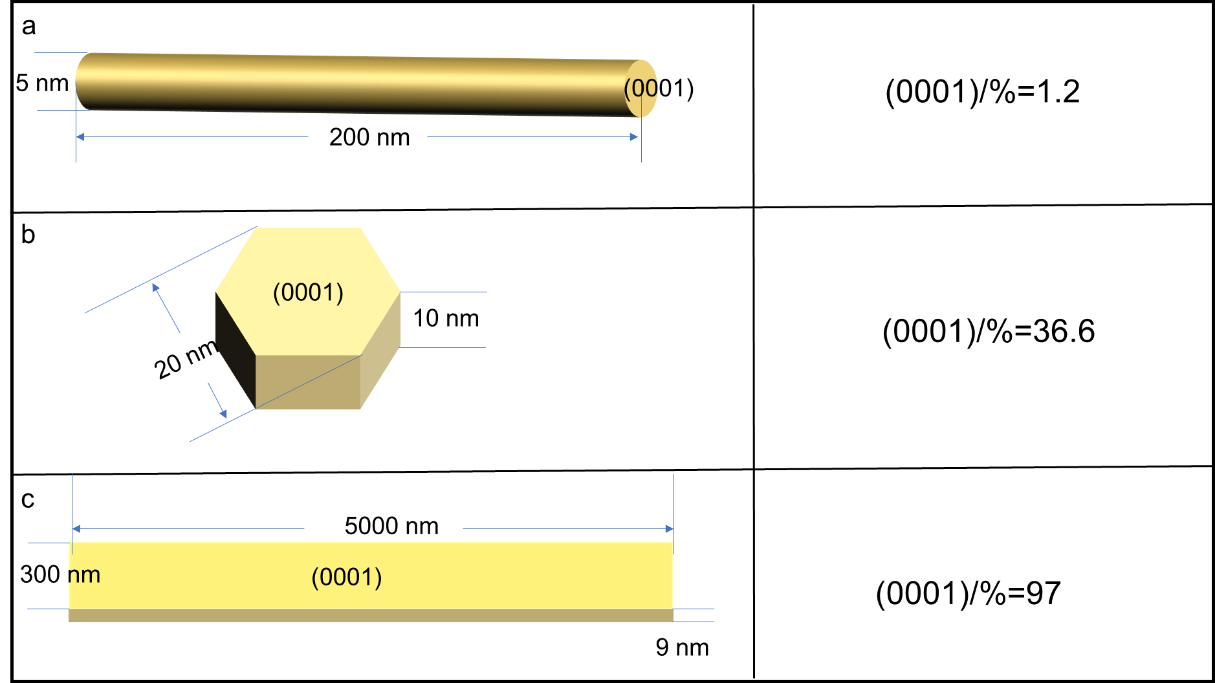


**Supplementary Figure 25 |** **The sketch maps of and ratios of (0001) facet**. **a**, nanorod. **b**, nanoparticle. **c**, nanobelt.


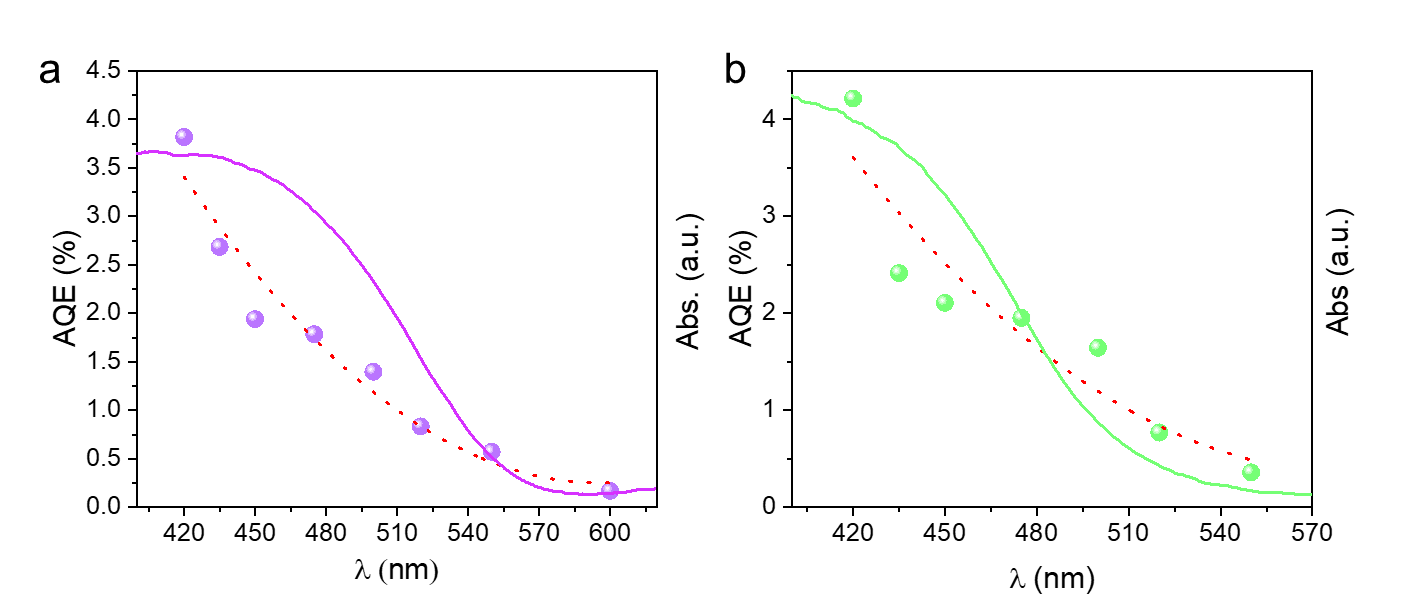


**Supplementary Figure 26 | Photocatalytic efficiency of the synthesized NBs**. **a**, Apparent quantum efficiency (AQE) in photocatalytic H_2_ evolution of CZIS NBs. **b**, AQE of CZGS NBs.


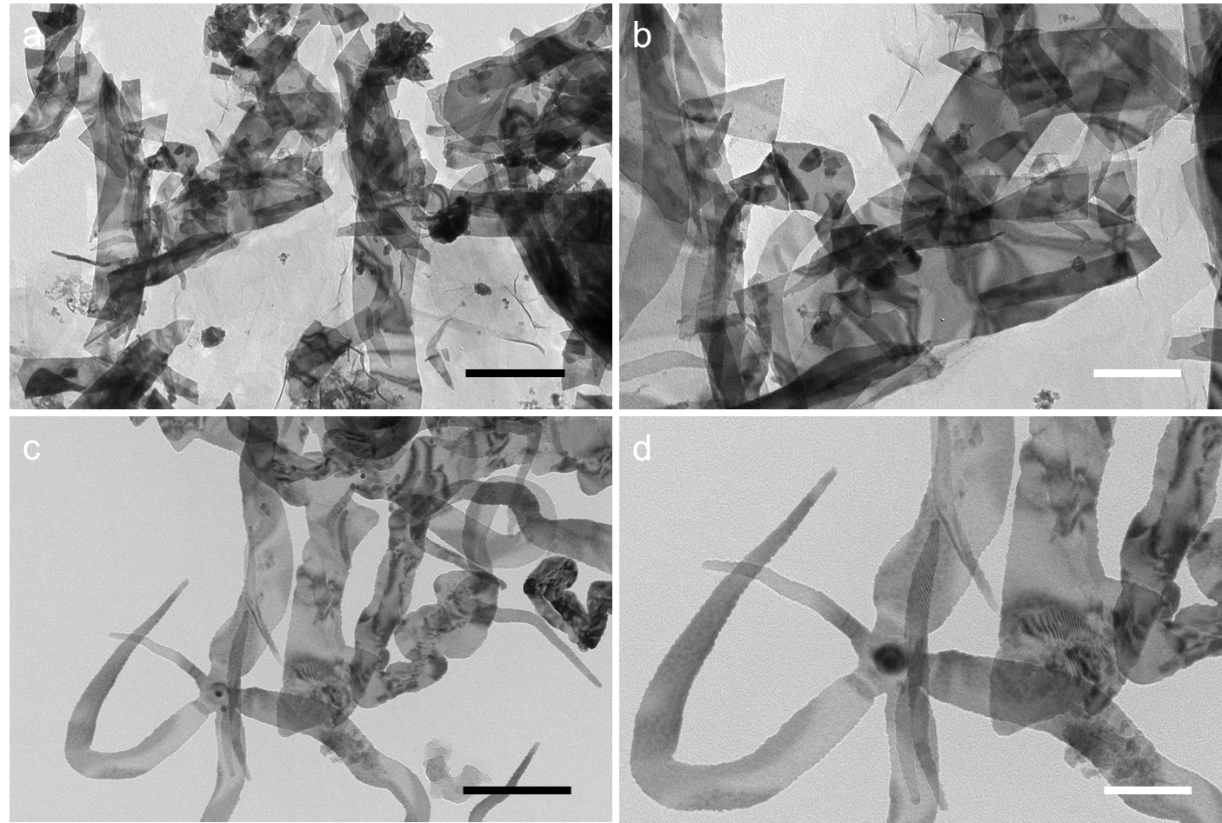


**Supplementary Figure 27 |** **TEM images of the NBs after six consecutive runs with each run of 4 h in photocatalytic test. a-b,** CZIS NBs. **c-d,** CZGS NBs. Scale bars are 500 nm for **a** and **c**, 200 nm for **b** and **d**, respectively.


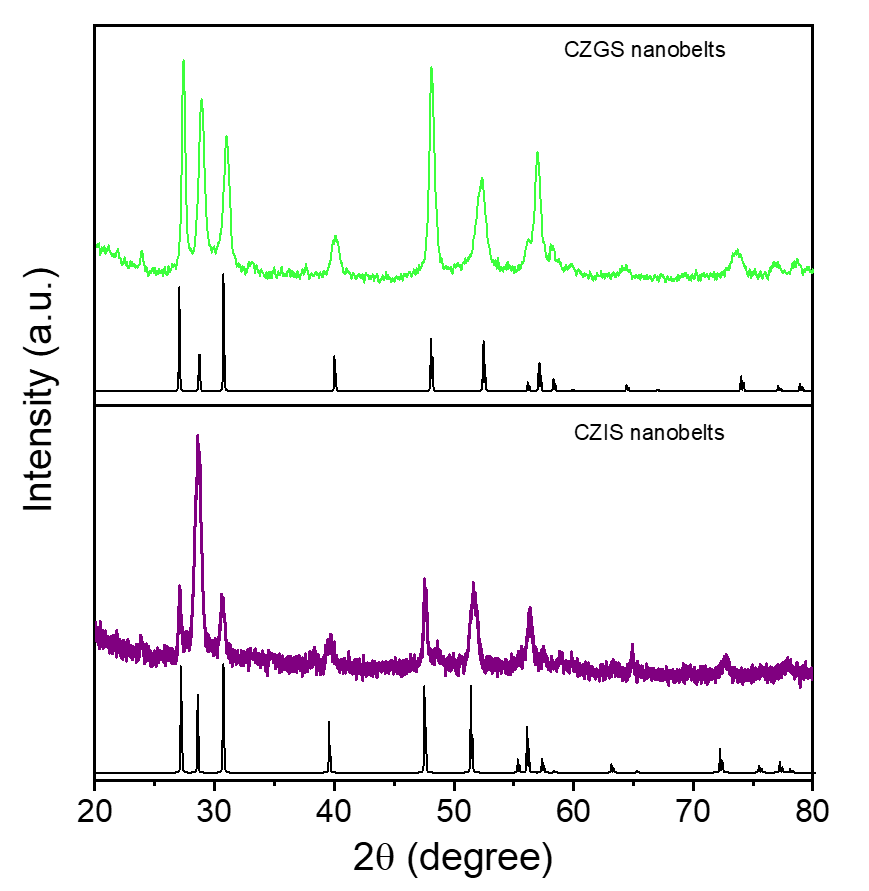


**Supplementary Figure 28 |** **XRD patterns of the CZIS and CZGS NBs after six consecutive runs with each run of 4 h in photocatalytic test**.


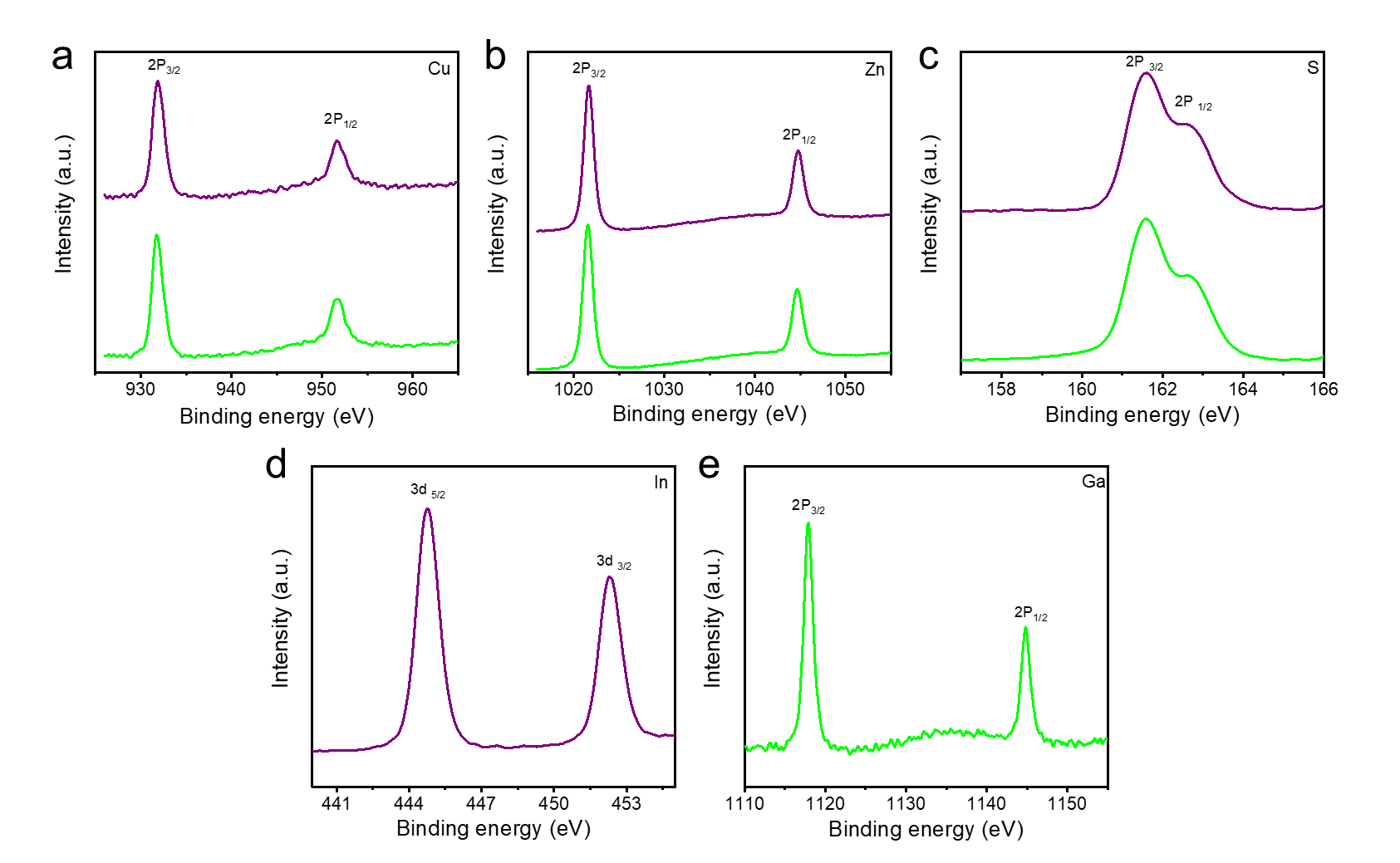


**Supplementary Figure 29 |** **XPS spectra of the CZIS and CZGS NBs after six consecutive runs**. **a,** Cu 2p. **b,** Zn 2P. **c,** S 2p. **d,** In 3d. **e,** Ga 2p.


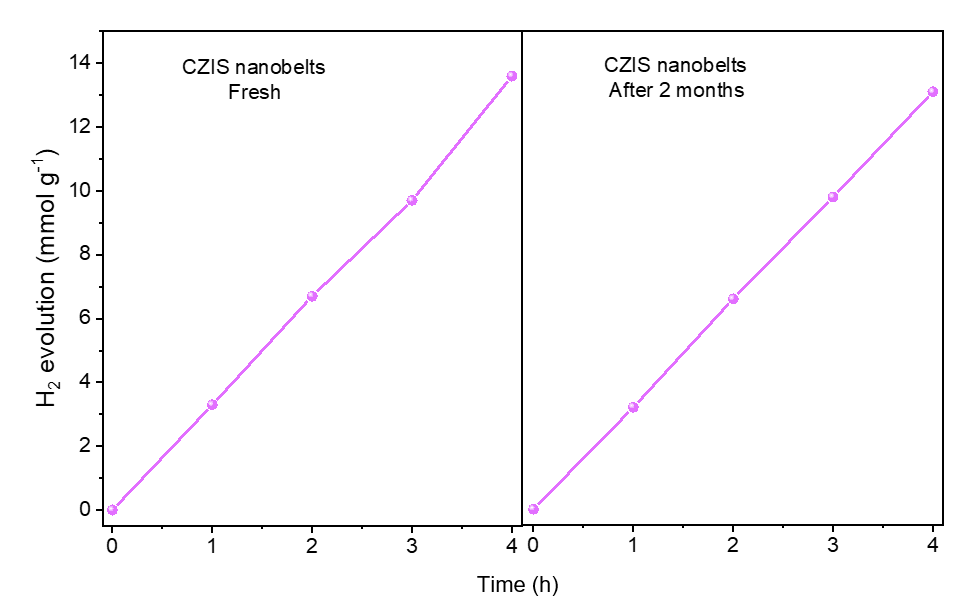


**Supplementary Figure 30 |** **Photoactivity test of fresh CZIS NBs and after storing under ambient conditions for two months**.


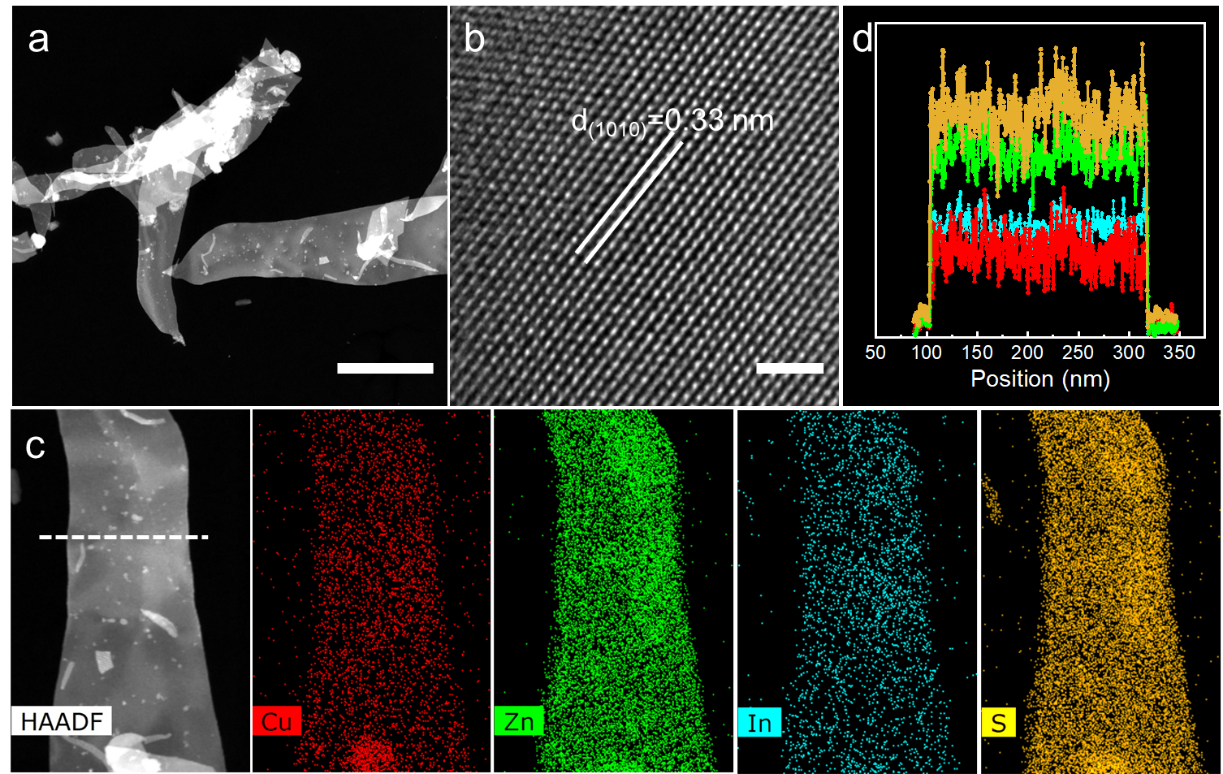


**Supplementary Figure 31 |** **Characterization of CZIS NBs stored under ambient conditions for two months and then after photocatalytic test.** **a,** HADDF image (The scale bar = 500 nm). **b,** HRTEM image (The scale bar = 2 nm). **c**, EDS-mapping. **d,** EDS- line scan. Scale bars are 500 nm for **a**, 2 nm for **b**, respectively.


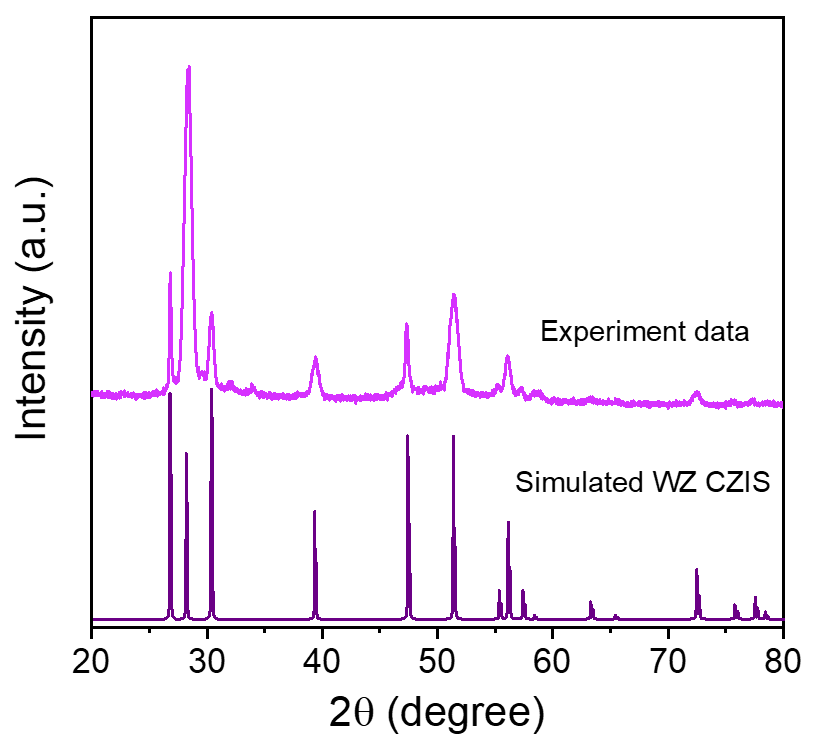


**Supplementary Figure 32 |** **XRD pattern of CZIS NBs stored under ambient conditions for two months and then after photocatalytic test**.


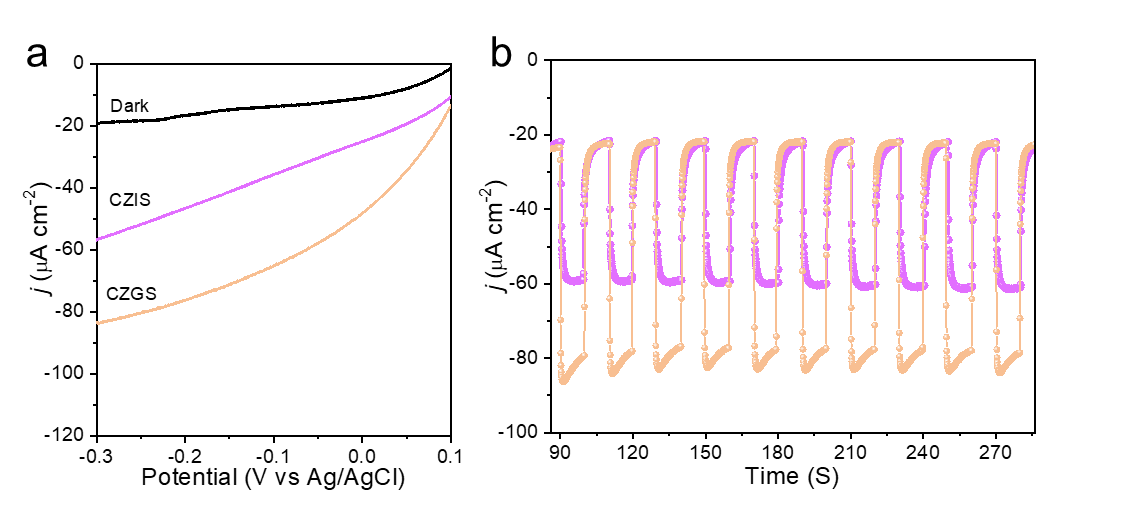


**Supplementary Figure 33 | Photoelectrochemical performance of CZIS and CZGS NBs. a,** Linear sweep voltammetry curves for PECs with different working electrodes. **b,** The photocurrent density-time response of CZIS (red) and CZGS (green) nanobelt ﬁlms at a potential of −0.25 V.

**Supplementary Table 1 | Comparison of the photocatalytic H_2_ production performances for the representative CZIS-based photocatalysts.**

| Photocatalyst | Hole scavenger | H_2_ (μmol/h/g) | Ref. |
| --- | --- | --- | --- |
| WZ-CZIS-NRs | 0.35 M Na_2_S and 0.25 M Na_2_SO_3_ | 70 | ^2^ |
| WZ-CZIS-NRs-Pt | 0.35 M Na_2_S and 0.25 M Na_2_SO_3_ | 250 | ^2^ |
| WZ-CZIS-NRs-Pd_4_S | 0.35 M Na_2_S and 0.25 M Na_2_SO_3_ | 180 | ^2^ |
| WZ-CZIS-SS-Ru | 0.35 M Na_2_S and 0.25 M K_2_SO_3_ | 200 | ^3^ |
| WZ-CZIS-HS-Ru | 0.35 M Na_2_S and 0.25 M K_2_SO_3_ | 360 | ^3^ |
| KS-CZIS-CQs | 0.35 M Na_2_S and 0.25 M Na_2_SO_3_ | 91.8 | ^4^ |
| KS-CZIS-CQs-Pt | 0.35 M Na_2_S and 0.25 M Na_2_SO_3_ | 456.4 | ^4^ |
| ZB-CZIS-HS-Rux | 0.35 M Na_2_S and 0.25 M Na_2_SO_3_ | 760 | ^5^ |
| ZB-CZIS-MS | 0.5 M Na_2_S and 0.5 M Na_2_SO_3_ | 91 | ^6^ |
| ZB-CZIS-NPs | 0.35 M Na_2_S and 0.25 M Na_2_SO_3_ | 984 | ^7^ |
| WZ-CZGS-NRs | 0.1 M Na_2_S and 0.1 M Na_2_SO_3_ | 730.2 | ^8^ |
| ZB-CZIS-NS | 0.1 M Na_2_S and 1.2 M Na_2_SO_3_ | 2100 | ^9^ |
| ZB-CZIS-NP-GO | 0.1 M Na_2_S and 1.2 M Na_2_SO_3_ | 3880 | ^9^ |
| CZIS-Pt | 0.35 M Na_2_S and 0.25 M K_2_SO_3_ | 684 | ^10^ |
| CZIS- NBs | 0.35 M Na_2_S and 0.25 M Na_2_SO_3_ | 3350 | **This work** |
| CZGS- NBs | 0.35 M Na_2_S and 0.25 M Na_2_SO_3_ | 3750 | **This work** |
| CdS-QDs | 0.35 M Na_2_S and 0.25 M Na_2_SO_3_ | 450 | ^11^ |
| CdS-NWs | 10% TEA | 79.3 | ^12^ |
| CdS-NSs | 0.35 M Na_2_S and 0.25 M Na_2_SO_3_ | 1540 | ^13^ |

**Supplementary Table 2 |** **Amounts of the precursors used for synthesizing the wurtzite CZIS NBs, the cation ratio and bandgap of the obtained CZIS NBs.**

| Sample | Cu# (mmol) | Zn# (mmol) | In#  (mmol) | Cu:Zn:In (EDS) | Bandgap  (eV) |
| --- | --- | --- | --- | --- | --- |
| In-1 | 0.1 | 0.1 | 0.1 | 1:0.7:0.8 | 1.55 |
| In-2 | 0.1 | 0.2 | 0.1 | 1:1.8:0.8 | 1.80 |
| In-3 | 0.1 | 0.3 | 0.1 | 1:2.9:1 | 2.11 |
| In-4 | 0.1 | 0.4 | 0.1 | 1:3.7:1.1 | 2.23 |
| In-5 | 0.1 | 0.5 | 0.1 | 1:4.5:1.1 | 2.37 |

**Supplementary Table 3 | Lattice constants of CZIS.**

| Facets | ΔE_H_ (eV) | TΔS_H_ (eV) | ΔE_ZEP_ (eV) | ΔG_H_ (eV) |
| --- | --- | --- | --- | --- |
| (0001) | 0.163 | -0.2 | 0.226 | 0.589 |
| (1010) | 1.091 | -0.2 | 0.215 | 1.506 |
| (1011) | 1.126 | -0.2 | 0.223 | 1.549 |

ΔG_H_ = ΔE_H_ + ΔE_ZPE_ - TΔS_H_

**Supplementary Table 4 | Lattice constants of CZIS.**

|  | a_0_ (Å) | b_0_ (Å) | c_0_ (Å) | c_0_/2a_0_ | *α*β*γ* |
| --- | --- | --- | --- | --- | --- |
| Experiment | 3.878 | 3.878 | 6.394 | 0.824 | 90*90*120 |
| Simulation | 3.855 | 3.855 | 6.328 | 0.820 | 90*90*120 |

**Supplementary Table 5 |** **Amounts of the precursors used for synthesizing the wurtzite CZGS NBs, the cation ratio and bandgap of the obtained CZGS NBs**

| Sample | Cu# (mmol) | Zn# (mmol) | Ga#  (mmol) | Cu:Zn:Ga (EDS) | Bandgap  (eV) |
| --- | --- | --- | --- | --- | --- |
| Ga-1 | 0.1 | 0.1 | 0.1 | 1:0.7:0.9 | 2.19 |
| Ga-2 | 0.1 | 0.2 | 0.1 | 1:1.4:0.8 | 2.31 |
| Ga-3 | 0.1 | 0.3 | 0.1 | 1:2.3:0.7 | 2.42 |
| Ga-4 | 0.1 | 0.4 | 0.1 | 1:3.5:0.8 | 2.48 |
| Ga-5 | 0.1 | 0.5 | 0.1 | 1:4.3:0.8 | 2.54 |

**Supplementary Methods**

**Materials.**

Copper (II) nitrate trihydrate (Cu(NO_3_)_2_·3H_2_O, 99%), Zinc nitrate hexahydrate (Zn(NO_3_)_2_·6H_2_O, 99%), Indium (III) nitrate hydrate (In(NO_3_)_3_·xH_2_O, 99.5%), Sodium diethyldithiocarbamate trihydrate (NaS_2_CNEt_2_, 99%). Ethanol (99.7%), Hexane (97%), 1-dodecanethiol (DDT, 97%), Oleic acid (OA, 99%), Chloroform were purchased from Sinopharm Chemical Reagent Co. Ltd (Shanghai). Europium (III) nitrate hexahydrate (Eu(NO_3_)_3_·6H_2_O, 99%) was purchased from Alfa Aesar. Gallium nitrate hydrate (Ga(NO_3_)_3_·xH_2_O, 99.99%), Sodium sulfide nonahydrate (Na_2_S·9H_2_O, 98%), Sodium sulfite (Na_2_SO_3_, 98%), Oleylamine (OLA, 80-90%) and 1-octadecene (ODE, 80-90%) were purchased from Aladdin Reagent Co. Ltd (Shanghai). All chemical reagents were used as received without further purification.

**Preparation of Cu(S_2_CNEt_2_)_2_ (Cu(dedc)_2_)**

NaS_2_CNEt_2_ (10 mmol) and Cu(NO_3_)_2_·3H_2_O (5 mmol) were dissolved in ionized water (100 ml), respectively. Then, Cu(NO_3_)_2_·3H_2_O aqueous solution was dropwise added to NaS_2_CNEt_2_ solution with vigorous stirring. After 30 min, the products are collected by centrifugation, washed at least 3 times with ionized water and ethanol, and dried in vacuum at 60 ^o^C.

**Preparation of Zn(dedc)_2_ (Zn#), In(dedc)_3_, Ga(dedc)_3_**

The synthesis method is the same as that of Cu(dedc)_2_.

**Synthesis of CZIS nanorods**

According to the reported method,^1^ Cu(dedtc)_2_ (0.1 mmol), In(dedtc)_3_ (0.1 mmol) and Zn(dedtc)_2_ (0.4 mmol) were dissolved in 4 mL of OLA, 4 mL of DDT and 3 mL of ODE in a three-neck flask in air. The next reaction procedure is in the same with the synthesis of CZIS NBs.

**Synthesis of CZIS nanoparticles**

In the synthesis of CZIS nanorods, Cu(dedtc)_2_ (0.1 mmol), In(dedtc)_3_ (0.1 mmol) and Zn(dedtc)_2_ (0.4 mmol) were dissolved in 10 mL of OLA in a three-neck flask in air. The reaction solution was heated up to 280 ^o^C at a heating rate of 10 ^o^C/min and kept at 280 ^o^C for 60 min under pure N_2._ The next reaction procedure is in the same with the synthesis of CZIS NBs.

**Ligands exchange**

The synthesized CZIS (50 mg) with hydrophobic ligands was dissolved in 5 mL chloroform (solution A). 0.5 g KOH was added to 15mL methanol with 0.5 mL MPA (solution B). Then, the solution A was swift added to the solution B. The mixing solution was stirred at room temperature for 5 hours. The NBs was collected by centrifugation and washed with water and methanol for twice. The final product was dispersed in water and stored in glovebox.

**Preparation of thin films of NBs**

The nanobelt thin films electrodes were prepared by dropping 100 μL aqueous dispersion with the sample concentration of 15 mg mL^-1^ onto a cleaned ITO glass, and the electrodes were annealed in vacuum for 12h at 150 ^o^C to remove the organic ligands.

**Supplementary References**

1. Ye, C., Regulacio, M. D., Lim, S. H., Xu, Q. H. & Han, M. Y. Alloyed (ZnS)_x_(CuInS_2_)_(1-x)_ semiconductor nanorods: synthesis, bandgap tuning and photocatalytic properties. *Chem. Eur. J.* **18**, 11258-11263 (2012).

2. Ye, C., Regulacio, M. D., Lim, S. H., Li, S., Xu, Q. H. & Han, M. Y. Alloyed ZnS-CuInS_2_ Semiconductor Nanorods and Their Nanoscale Heterostructures for Visible-Light-Driven Photocatalytic Hydrogen Generation. *Chem. Eur. J.* **21**, 9514-9519 (2015).

3. Huang, Y. *et al.* Enhanced photocatalytic hydrogen evolution efficiency using hollow microspheres of (CuIn)_(x)_Zn_(2(1-x))_S_2_ solid solutions. *Dalton Trans.* **44**, 10991-10996 (2015).

4. Tan, L. *et al.* Effective bandgap narrowing of Cu-In-Zn-S quantum dots for photocatalytic H_2_ production via cocatalyst-alleviated charge recombination. *Inorg. Chem. Front.* **5**, 258-265 (2018).

5. Chen, Y., Qin, Z., Guo, X., Wang, X. & Guo, L. One-step hydrothermal synthesis of (CuIn)_0.2_Zn_1.6_S_2_ hollow sub-microspheres for efficient visible-light-driven photocatalytic hydrogen generation. *Int. J. Hydrogen Energy* **41**, 1524-1534 (2016).

6. Lin, Y., Zhang, F. & Pan, D. A facile route to (ZnS)_x_(CuInS_2_)_1−x_ hierarchical microspheres with excellent water-splitting ability. *J. Mater. Chem.* **22**, 22619 (2012).

7. Xu, M., Zai, J., Yuan, Y. & Qian, X. Band gap-tunable (CuIn)_x_Zn_2(1−x)_S_2_ solid solutions: preparation and efficient photocatalytic hydrogen production from water under visible light without noble metals. *J. Mater. Chem.* **22**, 23929 (2012).

8. Liu, Z. *et al.* Non-injection synthesis of L-shaped wurtzite Cu-Ga-Zn-S alloyed nanorods and the advantageous application in photocatalytic hydrogen evolution. *J. Mater. Chem. A* **6**, 18649-18659 (2018).

9. Tang, X., Tay, Q., Chen, Z., Chen, Y., Goh, G. K. & Xue, J. CuInZnS-decorated graphene nanosheets for highly efficient visible-light-driven photocatalytic hydrogen production. *J. Mater. Chem. A* **1**, 6359-6365 (2013).

10. Tsuji, I., Kato, H., Kobayashi, H. & Kudo, A. Photocatalytic H_2_ Evolution under Visible-Light Irradiation over Band-Structure-Controlled (CuIn)_x_Zn_2(1-x)_S_2_ Solid Solutions. *J. Phys. Chem. B* **109**, 7323-7329 (2005).

11. Li, Y. *et al.* Pulsed axial epitaxy of colloidal quantum dots in nanowires enables facet-selective passivation. *Nat. Commun.* **9**, 4947 (2018).

12. Chen, J. *et al.* Edge Epitaxy of Two-Dimensional MoSe_2_ and MoS_2_ Nanosheets on One-Dimensional Nanowires. *J. Am. Chem. Soc.* **139**, 8653-8660 (2017).

13. Ye, L. *et al.* Robust and efficient photocatalytic hydrogen generation of ReS_2_/CdS and mechanistic study by on-line mass spectrometry and in situ infrared spectroscopy. *Appl. Catal. B: Environ.* **257**, 117897 (2019).
